# Development of 2-oindolin-3-ylidene-indole-3-carbohydrazide derivatives as novel apoptotic and anti-proliferative agents towards colorectal cancer cells

**DOI:** 10.1080/14756366.2020.1862100

**Published:** 2020-12-20

**Authors:** Wagdy M. Eldehna, Mahmoud F. Abo-Ashour, Tarfah Al-Warhi, Sara T. Al-Rashood, Amal Alharbi, Rezk R. Ayyad, Khayal Al-Khayal, Maha Abdulla, Hatem A. Abdel-Aziz, Rehan Ahmad, Radwan El-Haggar

**Affiliations:** a Department of Pharmaceutical Chemistry, Faculty of Pharmacy, Kafrelsheikh University, Kafrelsheikh, Egypt; b Department of Pharmaceutical Chemistry, Faculty of Pharmacy, Egyptian Russian University, Badr City, Egypt; c Department of Chemistry, College of Science, Princess Nourah bint Abdulrahman University, Riyadh, Saudi Arabia; d Department of Pharmaceutical Chemistry, College of Pharmacy, King Saud University, Riyadh, Saudi Arabia; e Department of Pharmaceutical Chemistry, Faculty of Pharmacy, Al-Azhar University, Cairo, Egypt; f Colorectal Research Chair, Department of Surgery, King Khalid University Hospital, King Saud University College of Medicine, Riyadh, Saudi Arabia; g Department of Applied Organic Chemistry, National Research Center, Giza, Egypt; h Pharmaceutical Chemistry Department, Faculty of Pharmacy, Helwan University, Cairo, Egypt

**Keywords:** Anti-proliferative indoles, apoptosis, isatin-indole, Bcl2/BclxL inhibitors, colorectal cancer, western blotting

## Abstract

Mitochondrial anti-apoptotic Bcl2 and BclxL proteins, are overexpressed in multiple tumour types, and has been involved in the progression and survival of malignant cells. Therefore, inhibition of such proteins has become a validated and attractive target for anticancer drug discovery. In this manner, the present studies developed a series of novel isatin–indole conjugates (**7a-j** and **9a-e**) as potential anticancer Bcl2 and BclxL inhibitors. The progression of the two examined colorectal cancer cell lines was significantly inhibited by all of the prepared compounds with IC_50_ ranges132–611 nM compared to IC_50_ = 4.6 µM for **5FU**, against HT-29 and IC_50_ ranges 37–468 nM compared to IC_50_ = 1.5 µM for **5FU**, against SW-620. Thereafter, compounds **7c** and **7g** were selected for further investigations. Interestingly, both compounds exhibited selective cytotoxicity against both cell lines with high safety to normal fibroblast (HFF-1). In addition, both compounds **7c** and **7g** induced apoptosis and inhibited Bcl2 and BclxL expression in a dose-dependent manner. Collectively, the high potency and selective cytotoxicity suggested that conjugates **7c** and **7g** could be a starting point for further optimisation to develop novel pro-apoptotic and antitumor agents towards colon cancer.

## 1. Introduction

Cancer is a group of diseases that is characterised by uncontrolled and rapid cell proliferation and differentiation mechanisms with the potential to invade or spread to other body parts^1^. Since several decades, cancer has been one of the main world health problems and is still considered a serious leading cause of death worldwide. Early strategies of cancer treatment were based on the unspecific induction of cell death mainly targeting the replication machinery^2–5^ and/or the DNA synthesis^6–9^. Therefore, the traditional anticancer drugs were associated with severe adverse effects due to the unselective toxicity towards the normal cells in addition to the resistance emerged towards them^9^. Thus, the development of effective and safe new antitumour drugs with increased selectivity towards cancer cells is still an active search^10^^,^^11^. On the other hand, recent strategies of targeted therapies target specific biomarkers essential for the regulation of cancer cells proliferation and/or cell apoptosis such as deregulated, mutated, or overexpressed proteins^12^ and thus, selectively affect cancer cells or their supporting environment with minimum effects on normal cells^13^. Among these targets are the pro-apoptotic and anti-apoptotic proteins that control the cellular apoptosis^14–16^.

Apoptosis, a form of programmed cell death takes place in multicellular organisms, is a series of biochemical events that result in characteristic cell changes and death^17^. Apoptosis could be launched through one of two pathways (intrinsic pathway and extrinsic pathway). The mitochondria-dependent apoptotic pathway (intrinsic pathway), one of the main pathways of induction of the cell apoptosis^18^^,^^19^, is controlled by the Bcl2 proteins family. The Bcl2 family members have dual functions; some are anti-apoptotic Bcl2 proteins, such as Bcl2 and BclxL that inhibit apoptosis, while others are pro-apoptotic Bcl2 proteins such as Bax and Bak that promote apoptosis^14^. In this regard, it was reported that most of cancer cells are characterised by over-expression of the anti-apoptotic Bcl2 proteins which could lead to apoptosis prevention as well as drug resistance^20^^,^^21^. Thus, the development of anti-apoptotic Bcl2 proteins inhibitors has become an important strategy for introducing potential anti-cancer agents^15^^,^^16^. In this manner, several heterocyclic scaffolds including isatin^22^ were developed as candidates targeting Bcl2 proteins.

**Figure 1.**
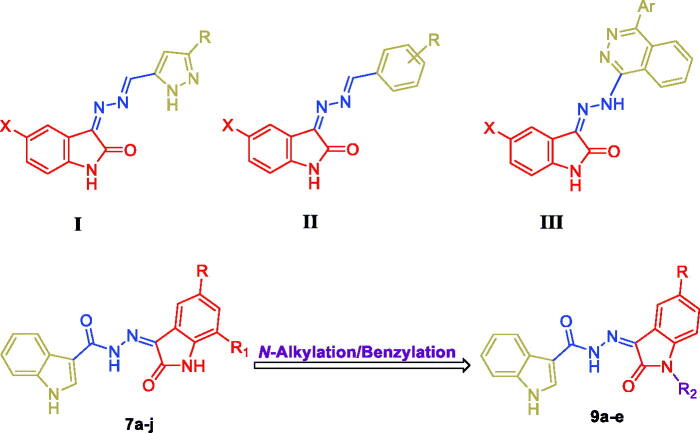
Chemical structures for some reported isatin-based anticancer conjugates (**I-III**), and the target conjugates (**7a-j** and **9a**-**e**).

Isatin (1*H*-indole-2,3-dione), as a special class in drug design and discovery, represents one of the most favourable scaffolds of heterocyclic systems which possesses many interesting biological activities including anti-SARS-CoV-2^23^, antimicrobial^24^, anticonvulsant^25^ and mainly anticancer^26–28^. Therefore, isatin nucleus was broadly used by our group for the development of diverse effective oxindole-based small molecules (structures **I**–**III**^29–31^, Figure 1) with anticancer activities that target different enzymatic and cellular targets such as inhibition of cancer-related carbonic anhydrase IX isoform^32–33^, inhibition of different kinases^34–35^, in addition to apoptosis induction in different human cancer cell lines^36–37^.

Motivated by the aforementioned findings and in continuation to our previous work, in the present study, a novel series of isatin/indole conjugates (**7a-j** and **9a-e**, Figure 1) were designed and synthesised. The antiproliferative effect of the new compounds against HT-29 and SW-620 colorectal cancer cell lines were examined. In addition, the levels of the mitochondria-related anti-apoptotic proteins Bcl2 and BclxL in HT-29 and SW-620 colorectal cancer cell lines after incubation with isatin derivatives **7c** and **7g** were determined.

## 2. Results and discussion

### 2.1. Chemistry

**Scheme 1.**
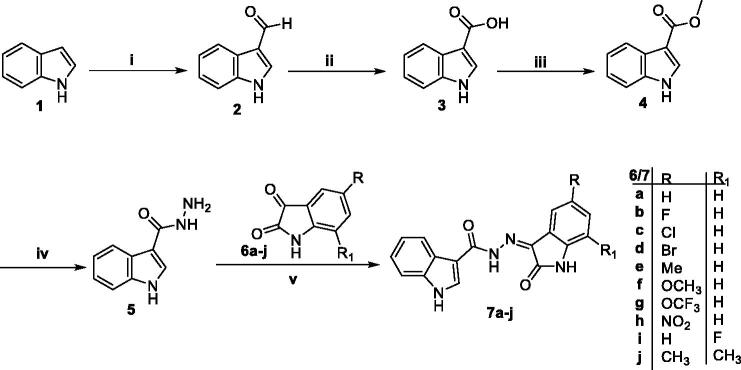
Synthesis of target 2-oxindolin-3-ylidene-indole-3-carbohydrazide **7a-j**; (**i**) DMF/POCl_3_/reflux 8 h, (**ii**) KMnO_4_/Acetone/Stirring at R.T 12 h, (**iii**) Dry methanol/H_2_SO_4_ (Cat.)/reflux 7 h, (**iv**) hydrazine hydrate/methanol/reflux 4 h, (**v**) Glacial acetic acid/reflux (5–7) h.

**Scheme 2.**
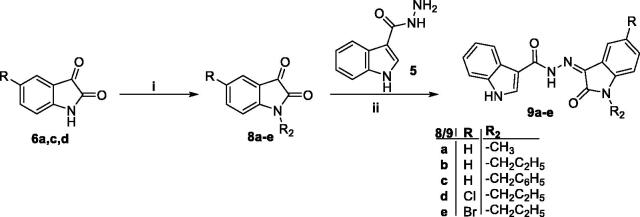
Synthesis of target *N*-substituted 2-oxindolin-3-ylidene-indole-3-carbohydrazide **9a-e**; (**i**) R-Br/DMF/K_2_CO_3_/KI (Cat.)/reflux 5 h, (**ii**) Glacial acetic acid/reflux (5–7) h.

The synthetic strategies deliberate for the development of the final compounds (**7a-j** and **9a-e**) were illustrated in Schemes 1–2. In Scheme 1, indole **1** was subjugated to formylation *via* Vilsmeier haack reaction to produce 1*H*-indole-3-carbaldehyde **2**, in which the CHO functionality was oxidised by KMnO_4_ in acetone to furnish 1*H*-indole-3-carboxylic acid **3**. Then, the acid analogue **3** was subjected to esterification through refluxing in dry methanol to get the carboxylate analogue **4**, where the ester group reacted with hydrazine hydrate in methyl alcohol to produce the key intermediate 1*H*-indole-3-carbohydrazide **5**. Finally, the key intermediate **5** was condensed with different isatin derivatives **6a-j** in glacial acetic acid to give the final targeted compounds **7a-j**.

On the other hand, in Scheme 2, three isatins **6a**, **6c** and **6d** were alkylated with methyl iodide, propyl bromide and benzyl bromide in DMF with the presence of K_2_CO_3_ and catalytic amount of KI to give the *N*-substituted isatin derivatives **8a-e**, which heated under reflux with the carbohydrazide **5** in acetic acid to furnish the final compounds **9a-e**, respectively. The structure of the synthesised 2-oxindolin-3-ylidene-indole-3-carbohydrazide was confirmed under the basis of spectral and elemental analyses which were in full agreement with the proposed structures.

### 2.2. Biological evaluation

#### 2.2.1. Anti-proliferative activity against HT-29 and SW-620 colorectal cancer cell lines

The anti-proliferative potential of the final compounds (**7a-j** and **9a-e**) was examined against two human colorectal cancer HT-29 and SW-620 cell lines. While, HT-29 is an adenocarcinoma cell line, SW-620 represents metastatic cancer cell line. These effects were compared with known anti-cancer drug, 5-Fluorouracil (**5FU**) commonly used in colorectal cancer treatment. All of the compounds were found to inhibit the cell viability of cancer cells with varied sub-micro-molar efficacy. The IC_50_ values ranged from 105 to 611 nM for all compounds were calculated using Graph Pad prism 8 (Table 1).

**Table 1.** *In vitro* anti-proliferative actions of compounds **7a-j** and **9a-e** towards HT-29 and SW-620 colorectal cancer cell lines.


Comp.	R	R_1_	R_2_	**IC_50_ (nM)**^a^	
				HT-29	SW-620
**7a**	H	H	H	408	145
**7b**	F	H	H	611	175
**7c**	Cl	H	H	206	188
**7d**	Br	H	H	320	133
**7e**	CH_3_	H	H	335	253
**7f**	OCH_3_	H	H	142	37
**7g**	OCF_3_	H	H	299	279
**7h**	NO_2_	H	H	132	190
**7i**	H	F	H	140	468
**7j**	CH_3_	CH_3_	H	200	187
**9a**	H	H	CH_3_	176	260
**9b**	H	H	propyl	525	105
**9c**	H	H	benzyl	405	283
**9d**	Cl	H	propyl	166	290
**9e**	Br	H	propyl	290	414
**5-FU**				4600	1500

^a^ IC_50_ values are the mean ± SD of three separate experiments.

Regarding the activity towards HT-29 cell line, all compounds showed superior activity to the reference drug (**5FU**) with IC_50_ ranged from 132-611 nM compared to IC_50_=4.6 µM for **5FU**. Compound **7h** was the most active against HT-29 cell line with IC_50_=132 nM that is approximately 35-fold more than **5FU**. Also, the results revealed that the *N*-alkylation of isatin moiety (series **9a-e**) resulted in diversified effect on the potency according to the alkyl group added and/or the substitution on indole moiety. For the unsubstituted indole (**7a**, IC_50_=408 nM), while *N*-alkylation with CH_3_- group significantly increase the potency (**9a**, IC_50_=176 nM) and *N*-alkylation with benzyl- group slightly increase the potency (**9c**, IC_50_=405 nM), the *N*-alkylation with propyl group dramatically decrease the potency (**9b**, IC_50_=525 nM). Furthermore, *N*-propylation of both chloro- (**7c**, IC_50_=206 nM) and bromo- (**7d**, IC_50_=320 nM) derivatives significantly increase the potency (**9d**, IC_50_=166 nM) and (**9e**, IC_50_=290 nM), respectively. Similarly, the activity towards SW-620 cell line of all compounds was more that **5FU** with IC_50_ ranged from 37–468 nM compared to IC_50_=1.5 µM for **5FU**. Compound **7f** was the most active against SW-620 cell line with IC_50_=37 nM that is approximately 32 folds more than **5FU**. However, *N*-alkylation of isatin moiety resulted in reduction of potency for all series except for compounds **9b** (IC_50_=105 nM) compared to compound **7a** (IC_50_=145 nM) (Table 1).

**Figure 2.**
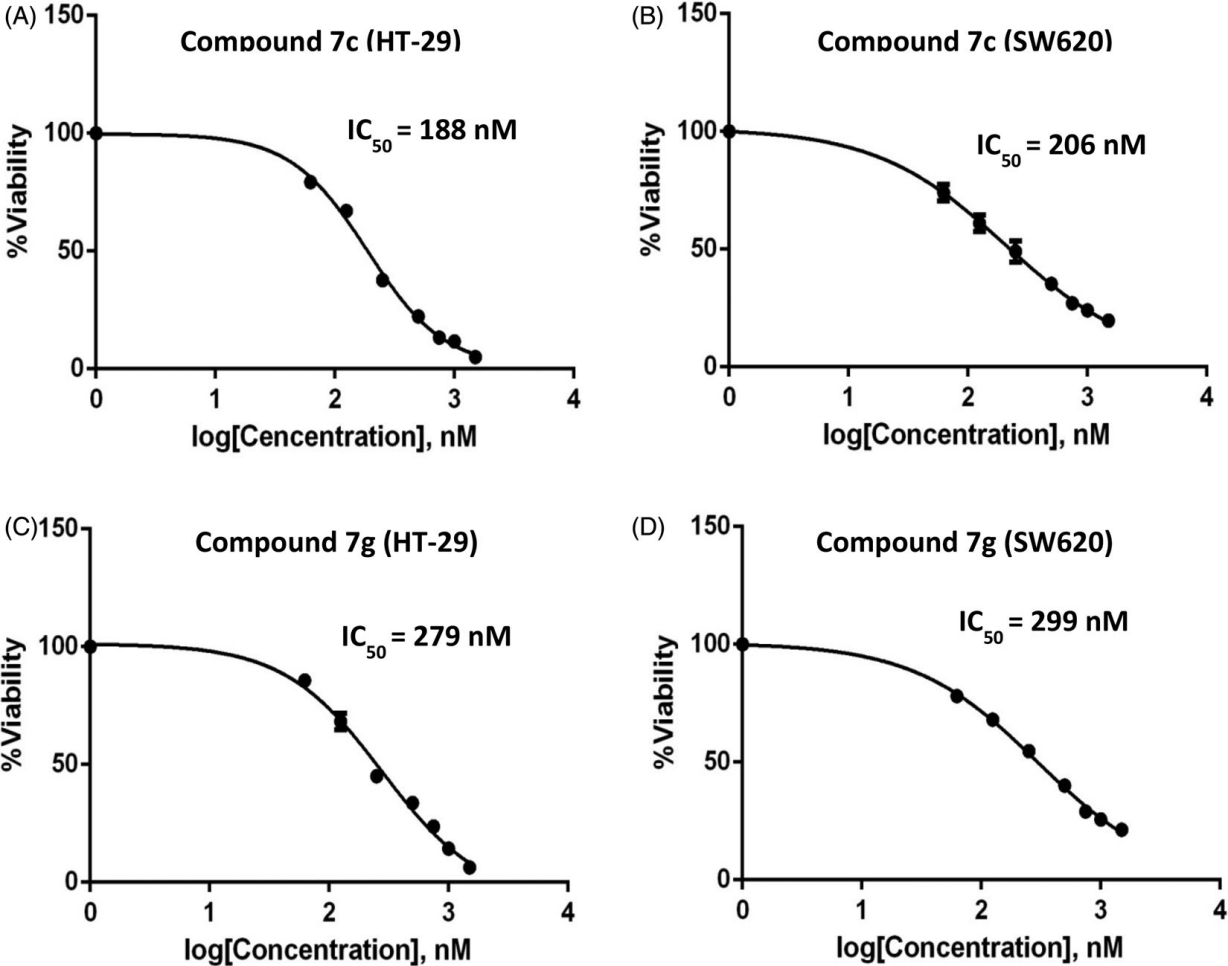
Effect of Compound **7c** and **7g** on the cell viability. Different concentrations of compound **7c** and **7g** have been used to study its effect on cell viability by MTT assay. Results of percent viability vs concentration were plotted and the IC_50_ was calculated for each cell line using Graph Pad prism 8. (A) HT-29 with compound **7c**, (B) SW-620 with compound **7c**, (C) HT-29 with compound **7g**, and (D) SW-620 with compound **7g**.

Figure 2 illustrated that treatment of HT-29 and SW-620 cell lines with different concentrations of compounds **7c** and **7g** resulted in a dose-dependent inhibition of cell viability. Compound **7c** was found to inhibit HT-29 and SW-620 with IC_50_=188 nM and 206 nM, respectively (Figure 2(A,B)), whereas compound **7g** was found to exhibit IC_50_ of 279 nM against HT-29 and 299 nM against SW-620 cell lines (Figure 2(C,D)), compared to the IC_50_ for **5FU** that was found to be 1.5 µM against HT-29 and 4.6 µM against SW-620 cell lines.

**Figure 3.**
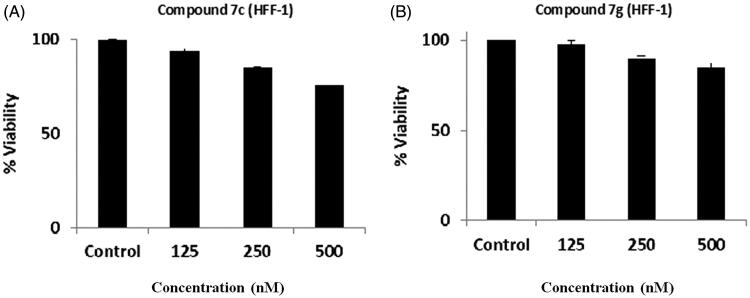
Effect of Compounds **7c** and **7g** on HFF-1. HFF-1 fibroblast cells were treated with compound **7c** and **7g** for 24 h and % viability was measured using MTT assay. (A) HFF-1 with compound **7c** and (B) HFF-1 with compound **7g**.

Furthermore, to investigate the selective cytotoxicity of the tested compounds, the effect of compounds **7c** and **7g** was studied on normal human skin fibroblast (HFF-1). Compounds **7c** and **7g** were found to have no/or little effect on cell viability of HFF-1 (Figure 3(A,B)). This finding indicates that compounds **7c** and **7g** inhibited cell viability of cancer cells without affecting normal fibroblast.

#### 2.2.2. Annexin V-FITC apoptosis assay

**Figure 4.**
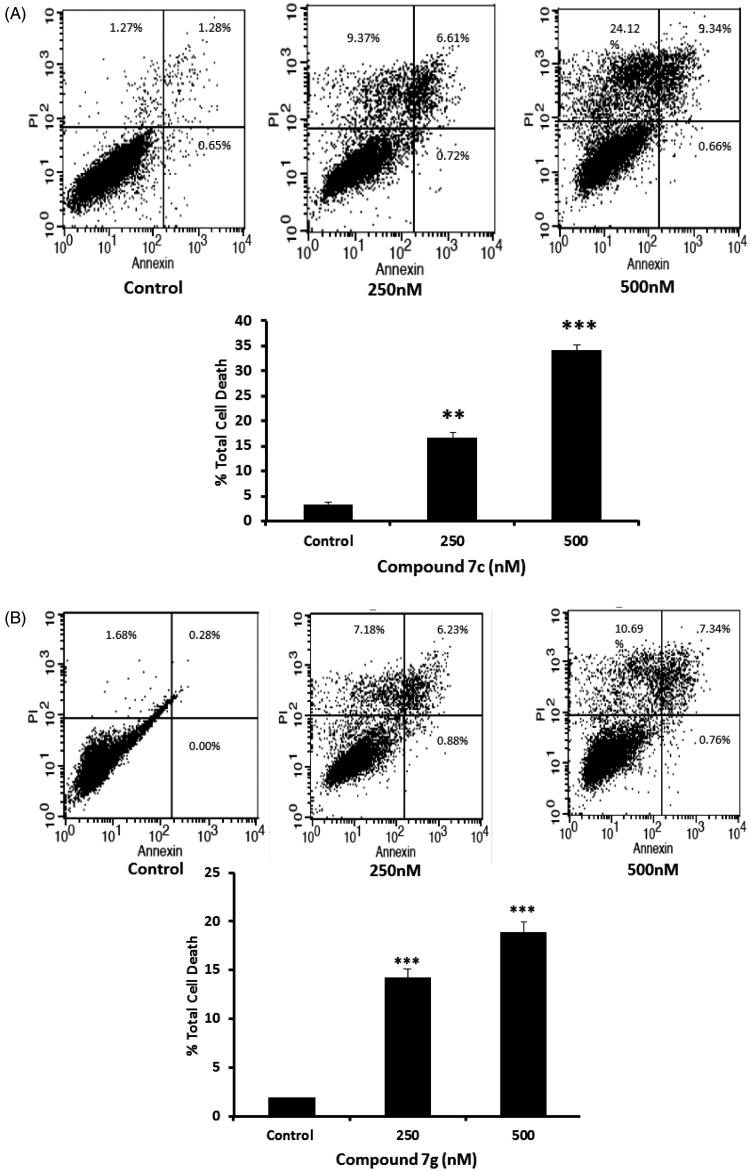
(A) Annexin V apoptosis assay for compound **7c**. SW-620 cells were treated with compound **7c** (250 and 500 nM) for 24 h. Quadrant was set for the resulted dots population from the results of the negative auto-fluorescence and the statistical analysis was performed where the significance of data was assessed at *p* values* < 0.05*. **p < 0.01; ***p < 0.001 control vs treated. (B) Annexin V apoptosis assay for compound **7g**. SW-620 cells were treated with compound **7g** (250 and 500 nM) for 24 h. Quadrant was set for the resulted dots population from the results of the negative auto-fluorescence and the statistical analysis was performed where the significance of data was assessed at *p* values* < 0.05*. **p < 0.01; ***p < 0.001 control vs treated.

Induction of apoptosis is mainly considered as an important strategy for the development of new anti-proliferative agents^38^. Compounds **7c** and **7g** were further investigated for their potential role of apoptosis induction in the colorectal SW-620 cancer cell line (SW-620 cell line was selected because most of the new compounds showed significant potency more than HT-29 cell line). Exposure of SW-620 cells to compounds **7c** and **7g** resulted in the induction of apoptosis in a dose dependent manner (Figure 4). As shown in the results, compound **7c** at concentrations of 250 nM and 500 nM was able to induce approximately 5.2- and 10.66-fold, respectively, total apoptosis increase compared to the control for SW-620 cell line (Figure 4(A)). Similarly, compound **7g** induced approximately 7.3 and 9.6 fold total apoptosis increase compared to the control when incubated with SW-620 cell line at concentration of 250 nM and 500 nM, respectively (Figure 4(B)). These findings encouraged us to further investigate the effect of compounds **7c** and **7g** towards the anti-apoptotic mitochondrial markers Bcl2 and BclxL.

#### 2.2.3. Impact of compounds 7c and 7g on the anti-apoptotic (Bcl2, and BclxL) markers levels

**Figure 5.**
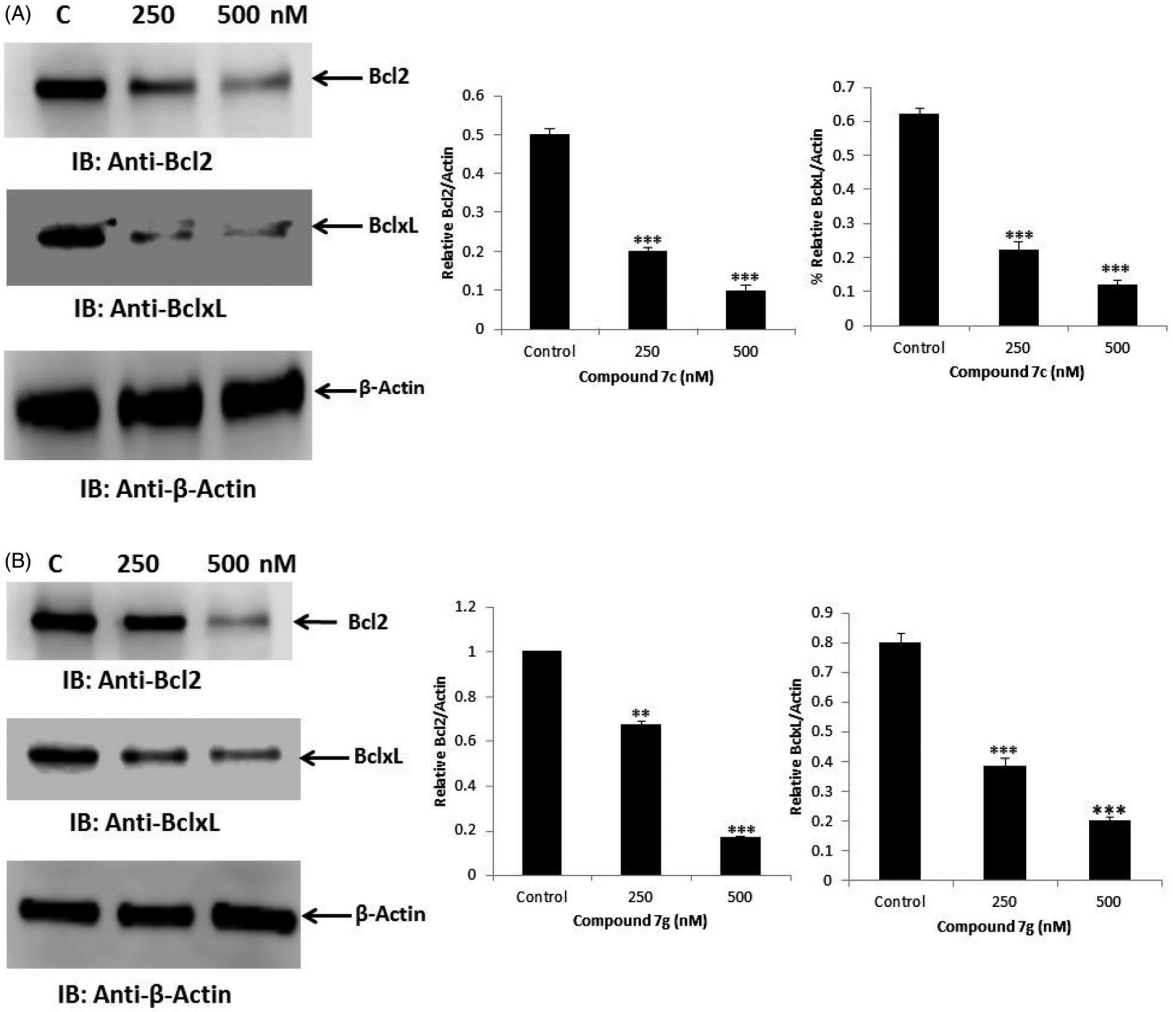
(A) Effect of compound **7c** on anti-apoptotic proteins. SW-620 cancer cells were treated with two concentrations of compound **7c** for 24 h. Immunoblotting was performed with the indicated antibodies. The band densities were measured and the bar charts were created to compare the effect on proteins. The statistical analysis was performed where the significance of data was assessed at *a p* values* < 0.05*. *** *p* < 0.001; ** *p* < 0.01 control vs treated. (B) Effect of compound **7g** on anti-apoptotic proteins. SW-620 cancer cells were treated with two concentrations of compound **7g** for 24 h. Immunoblotting was performed with the indicated antibodies. The band densities were measured and the bar charts were created to compare the effect on proteins.

Bcl2 and BclxL, as anti-apoptotic proteins, are known to be overexpressed in diverse tumours causing cancer cell survival and drug resistance^14^. Inhibition of these proteins expression resulted in cancer cell death and has been exploited as a strategy for anticancer drug discovery^15–16^. Treatment of SW-620 with compound **7c** resulted in a dose-dependent inhibition of Bcl2 and BclxL protein expression (Figure 5(A)). Compound **7g** was also found to inhibit Bcl2 and BclxL expression in SW-620 cells (Figure 5(B)). These findings thus indicate that compound **7c** and **7g** inhibited cell viability by inhibiting Bcl2 and BclxL resulting in the apoptosis.

## 3. Conclusions

In the present work, a series of novel isatin-indole conjugates (**7a-j** and **9a-e**) was designed and synthesised as potential antiproliferative agents towards colon cancer cells with promising inhibitory activity against the anti-apoptotic Bcl2 and BclxL proteins. The cell growth of two examined colorectal cancer (HT-29 and SW-620) cell lines was significantly inhibited by all of the prepared compounds with IC_50_ ranges 132–611 nM against HT-29 and IC_50_ ranges 37–468 nM against SW-620 as compared to IC_50_=4.6 and 1.5 µM for **5FU**, respectively. For further mechanistic and selective cytotoxicity studies, compounds **7c** and **7g** were examined and proved to exhibit selective cytotoxicity against both cancer cell lines with high safety profile to normal fibroblast (HFF-1). In addition, towards SW-620 cell line, both candidates inducted apoptosis and inhibited anti-apoptotic Bcl2 and BclxL proteins in a dose dependent manner. Collectively, the high potency and selective cytotoxicity suggested that conjugates **7c** and **7g** could serve as lead compounds for further optimisation to develop novel antitumor agents and Bcl2/BclxL inhibitors.

## 4. Experimental

### 4.1. Chemistry

#### 4.1.1. General

The NMR spectra have been recorded by Bruker spectrometer at 400 MHz. ^13 ^C NMR spectra were run at 100 MHz in deuterated dimethylsulphoxide (DMSO-*d6*). Chemical shifts (*δ_H_*) are reported relative to the solvent (DMSO-*d_6_*). Infra-red spectra were recorded on Schimadzu FT-IR 8400S spectrophotometer. Elemental analyses have been performed at the Regional Centre for Microbiology and Biotechnology, Al-Azhar University, Cairo, Egypt.

#### 4.1.2. Synthesis of the target compounds 7a-j and 9a-e

To hot stirred solution of 1*H*-indole-3-carbohydrazide **5** (0.3 g, 1.7 mmole) in 15 ml of glacial acetic acid, an equivalent amount of appropriate isatin derivatives **6a-j** and **8a-e** was added. The reaction mixture was heated under reflux for (5–7) h, and then was cooled to room temperature. The formed precipitate was collected by filtration, washed with cold water, hexane and recrystallized from DMF-MeOH mixture to furnish the targeted novel compounds **7a-j** and **9a-e**, respectively. Representative NMR spectra charts were provided in the Supplementary data.

##### 4.1.2.1. N'-(2-Oxoindolin-3-ylidene)-1H-indole-3-carbohydrazide 7a

Yellow powder; m.p. 259–261 °C; (yield 81%); ^1^H NMR *δ ppm*: 6.93 (d, 0.3H, Ar-H, *J* = 8.0 Hz), 6.98 (d, 0.7H, Ar-H, *J* = 8.0 Hz), 7.07–7.16 (*m*, 1H, Ar-H), 7.22–7.29 (*m*, 2H, Ar-H), 7.37 (*t*, 1H, Ar-H, *J* = 8.0 Hz), 7.52–7.58 (*m*, 1H, Ar-H), 7.65 (d, 0.7H, Ar-H, *J* = 8.0 Hz), 8.13 (d, 0.3H, Ar-H, *J* = 8.0 Hz), 8.23–8.32 (*m*, 1.7H, Ar-H), 8.67 (*s*, 0.3H, Ar-H), 10.83, 13.45 (*s*, 1H, NH of isatin), 11.18, 11.30 (*s*, 1H, hydrazide NH), 12.01, 12.05 (*s*, 1H, indole NH); ^13 ^C NMR *δ ppm*: 107.50, 108.17, 110.96, 111.56, 112.56, 112.99, 116.14, 120.75, 120.96, 120.98, 121.73, 121.79, 121.97, 122.21, 123.03, 123.21, 126.19, 126.54, 127.75, 131.51, 132.48, 133.62, 136.22, 136.65, 136.78, 142.41, 143.91 (Aromatic carbons), 162.16, 163.37 (C=O hydrazide), 165.27, 165.65 (C=O isatin)); IR (KBr, *ν* cm^−1^) 3504, 3280, 3245 (3NH) and 1707, 1654 (2 C=O); Calcd. Anal. for C_17_H_12_N_4_O_2_: C, 67.10; H, 3.97; N, 18.41; found C, 66.83; H, 4.57; N, 18.41.

##### 4.1.2.2. N'-(5-Fluoro-2-oxoindolin-3-ylidene)-1H-indole-3-carbohydrazide 7b

Red powder; m.p. >300 °C; (yield 78%); ^1^H NMR *δ ppm*: 6.90–9.93 (*m*, 0.7H, Ar-H), 6.96–7.00 (*m*, 0.3H, Ar-H), 7.20–7.30 (*m*, 3H, Ar-H), 7.49–7.58 (*m*, 1.3H, Ar-H), 8.15 (d, 0.7H, Ar-H, *J* = 8.0 Hz), 8.23 (d, 0.3H, Ar-H, *J* = 8.0 Hz), 8.31 (d, 1H, Ar-H, *J* = 8.0 Hz), 8.72 (*s*, 0.7H, Ar-H), 10.84, 13.41 (*s*, 1H, NH of isatin), 11.31, 11.35 (*s*, 1H, hydrazide NH), 12.03 (*s*, 1H, indole NH); IR (KBr, *ν* cm^−1^) 3659, 3460, 3285 (3NH) and 1712, 1669 (2C=O); Calcd. Anal. for C_17_H_11_FN_4_O_2_: C, 63.35; H, 3.44; N, 17.38; found C, 63.14; H, 4.01; N, 17.37.

##### 4.1.2.3. N'-(5-Chloro-2-oxoindolin-3-ylidene)-1H-indole-3-carbohydrazide 7c

Yellow powder; m.p. >300 °C; (yield 75%); ^1^H NMR *δ ppm*: 6.92 (d, 0.7H, Ar-H, *J* = 8.4 Hz), 6.97 (d, 0.3H, Ar-H, *J* = 8.4 Hz), 7.20–7.29 (*m*, 2H, Ar-H), 7.39–7.43 (*m*, 1H, Ar-H), 7.51–7.58 (*m*, 1H, Ar-H), 7.64 (*s*, 0.3H, Ar-H), 8.22–8.35 (*m*, 2H, Ar-H), 8.71 (*s*, 0.7H, Ar-H), 10.95, 12.03 (*s*, 1H, NH of isatin), 11.43 (*s*, 1H, hydrazide NH), 12.03 (*s*, 1H, Indole NH); IR (KBr, *ν* cm^−1^) 3593, 3429, 3331 (3NH) and 1720, 1643 (2 C=O); Calcd. Anal. for C_17_H_11_ClN_4_O_2_: C, 60.28; H, 3.27; N, 16.54; found C, 59.77; H, 3.88; N, 16.37.

##### 4.1.2.4. N'-(5-Bromo-2-oxoindolin-3-ylidene)-1H-indole-3-carbohydrazide 7d

Orange powder; m.p. 247–249 °C; (yield 83%); ^1^H NMR *δ ppm*: 6.91 (d, 1H, Ar-H, *J* = 8.0 Hz), 7.20–7.29 (*m*, 2H, Ar-H), 7.51 (d, 1H, Ar-H, *J* = 8.0 Hz), 7.55 (d, 1H, Ar-H, *J* = 8.0 Hz), 7.73 (*s*, 1H, Ar-H), 8.23 (d, 1H, Ar-H, *J* = 8.0 Hz), 8.34 (*s*, 1H, Ar-H), 11.34 (*s*, 1H, NH of isatin), 12.04 (*s*, 1H, hydrazide NH), 13.31 (*s*, 1H, Indole NH); ^13 ^C NMR *δ ppm*: 107.76, 111.54, 112.97, 113.46, 114.78, 121.03, 122.02, 122.86, 123.24, 126.36, 133.58, 133.92, 136.70, 141.39, 142.39 (Aromatic carbons), 162.99 (C=O hydrazide), 163.35 (C=O isatin)); IR (KBr, *ν* cm^−1^) 3621, 3428, 3184 (3NH) and 1710, 1638 (2 C=O); Calcd. Anal. for C_17_H_11_BrN_4_O_2_: C, 53.28; H, 2.89; N, 14.62; found C, 52.79; H, 2.82; N, 14.63.

##### 4.1.2.5. N'-(5-Methyl-2-oxoindolin-3-ylidene)-1H-indole-3-carbohydrazide 7e

Red powder; m.p. 201–203 °C; (yield 74%); ^1^H NMR *δ ppm*: 1.93, 2.33 (*s*, 3H, CH_3_), 6.85 (d, 1H, Ar-H, *J* = 8.0 Hz), 7.16 (d, 1H, Ar-H, *J* = 8.0 Hz), 7.22–7.29 (*m*, 2H, Ar-H), 7.45 (*s*, 1H, Ar-H), 7.55 (d, 1H, Ar-H, *J* = 8.0 Hz), 8.23–8.27 (*m*, 2H, Ar-H), 11.17 (*s*, 1H, NH of isatin), 12.04 (*s*, 1H, hydrazide NH), 13.44 (*s*, 1H, Indole NH)); IR (KBr, *ν* cm^−1^) 3451, 3322, 3252 (3NH) and 1711, 1660 (2 C=O); Calcd. Anal. for C_18_H_14_N_4_O_2_: C, 67.92; H, 4.43; N, 17.60; found C, 67.25; H, 4.98; N, 17.57.

##### 4.1.2.6. N'-(5-Methoxy-2-oxoindolin-3-ylidene)-1H-indole-3-carbohydrazide 7f

Red powder; m.p. 197–199 °C; (yield 80%); ^1^H NMR *δ ppm*: 3.81(*s*, 3H, OCH_3_), 6.89 (d, 1H, Ar-H, *J* = 8.0 Hz), 6.95 (d, 1H, Ar-H, *J* = 8.0 Hz), 7.19 (*s*, 1H, Ar-H), 7.22–7.29 (*m*, 2H, Ar-H), 7.55 (d, 1H, Ar-H, *J* = 8.0 Hz), 8.22–8.26 (*m*, 2H, Ar-H), 11.11 (*s*, 1H, NH of isatin), 12.05 (*s*, 1H, hydrazide NH), 13.52 (*s*, 1H, Indole NH); ^13 ^C NMR *δ ppm*: 56.09 (OCH3), 106.08, 108.20, 112.35, 112.99, 117.66, 120.94, 121.51, 121.96, 123.21, 126.12, 131.77, 136.07, 136.79, 155.87 (Aromatic carbons), 162.04 (C=O hydrazide), 163.51 (C=O isatin)); IR (KBr, *ν* cm^−1^) 3569, 3432, 3174 (3NH) and 1686, 1655 (2 C=O); Calcd. Anal. for C_18_H_14_N_4_O_3_: C, 64.67; H, 4.22; N, 16.76; found C, 64.09; H, 4.76; N, 16.56.

##### 4.1.2.7. N'-(2-Oxo-5-(trifluoromethoxy)indolin-3-ylidene)-1H-indole-3-carbohydrazide 7g

Yellow powder; m.p. 278–280 °C; (yield 72%); ^1^H NMR *δ ppm*: 7.05 (d, 1H, Ar-H, *J* = 8.0 Hz), 7.23–7.29 (*m*, 2H, Ar-H), 7.36 (d, 1H, Ar-H, *J* = 8.0 Hz), 7.55–7.60 (*m*, 2H, Ar-H), 8.22 (d, 1H, Ar-H, *J* = 8.0 Hz), 8.31 (*s*, 1H, Ar-H), 11.46 (*s*, 1H, NH of isatin), 12.06 (*s*, 1H, hydrazide NH), 13.39 (*s*, 1H, Indole NH)); IR (KBr, *ν* cm^−1^) 3647, 3445, 3187 (3NH) and 1710, 1641 (2 C=O); Calcd. Anal. for C_18_H_11_F_3_N_4_O_3_: C, 55.68; H, 2.86; N, 14.43; found C, 55.53; H, 2.91; N, 14.39.

##### 4.1.2.8. N'-(5-Nitro-2-oxoindolin-3-ylidene)-1H-indole-3-carbohydrazide 7h

Yellow powder; m.p. >300 °C; (yield 86%); ^1^H NMR *δ ppm*: 7.12 (d, 1H, Ar-H, *J* = 8.0 Hz), 7.22–7.29 (*m*, 2H, Ar-H), 7.54 (d, 1H, Ar-H, *J* = 8.0 Hz), 8.20 (d, 1H, Ar-H, *J* = 8.0 Hz), 8.24 (d, 1H, Ar-H, *J* = 8.0 Hz), 8.30–8.34 (*m*, 2H, Ar-H), 11.89 (*s*, 1H, NH of isatin), 12.11 (*s*, 1H, hydrazide NH), 13.19 (*s*, 1H, Indole NH); ^13 ^C NMR *δ ppm*: 107.64, 111.74, 113.00, 115.84, 120.96, 121.46, 121.92, 122.10, 123.31, 127.21, 132.40, 133.29, 136.71, 143.24, 147.41 (Aromatic carbons), 163.65 (C=O hydrazide), 166.06 (C=O isatin)); IR (KBr, *ν* cm^−1^) 3594, 3426, 3111 (3NH) and 1704, 1671 (2 C=O); Calcd. Anal. for C_17_H_11_N_5_O_4_: C, 58.46; H, 3.17; N, 20.05; found C, 57.91; H, 3.71; N, 20.08.

##### 4.1.2.9. N'-(7-Fluoro-2-oxoindolin-3-ylidene)-1H-indole-3-carbohydrazide 7i

Yellow powder; m.p. >300 °C; (yield 79%); ^1^H NMR *δ ppm*: 7.08–7.17 (m, 1H, Ar-H), 7.21–7.29 (*m*, 2H, Ar-H), 7.31 (*t*, 1H, Ar-H, *J* = 8.0 Hz), 7.50–7.58 (*m*, 1H, Ar-H), 8.03 (d, 1H, Ar-H, *J* = 8.0 Hz), 8.31 (d, 1H, Ar-H, *J* = 8.0 Hz), 8.68 (*s*, 1H, Ar-H), 11.29 (*s*, 1H, NH of isatin), 12.05 (*s*, 1H, hydrazide NH), 13.43 (*s*, 1H, Indole NH); ^13 ^C NMR *δ ppm*: 107.34, 108.02, 112.57, 113.01, 117.04, 118.17, 118.68, 119.11, 119.28,120.96, 121.72, 122.03, 122.56, 122.97, 123.27, 123.70, 123.92, 126.20, 127.76, 129.12, 129.25, 130.80, 131.97, 133.86, 136.21, 136.78, 145.81, 146.21, 148.22, 148.63 (Aromatic carbons), 162.09, 163.17 (C=O hydrazide), 165.30, 165.44 (C=O isatin)); IR (KBr, *ν* cm^−1^) 3527, 3450, 3286 (3NH) and 1716, 1638 (2 C=O); Calcd. Anal. for C_17_H_11_FN_4_O_2_: C, 63.35; H, 3.44; N, 17.38; found C, 63.11; H, 4.08; N, 17.33.

##### 4.1.2.10. N'-(5,7-Dimethyl-2-oxoindolin-3-ylidene)-1H-indole-3-carbohydrazide 7j

Red powder; m.p. >300 °C; (yield 74%); ^1^H NMR *δ ppm*: 2.22 (*s*, 3H, CH_3_ of position 7 for isatin), 2.30 (*s*, 3H, CH_3_ of position 5 for isatin), 7.00 (*s*, 1H, Ar-H), 7.15–7.28 (*m*, 3H, Ar-H), 7.54 (d, 1H, Ar-H, *J* = 8.0 Hz), 8.23–8.34 (*m*, 2H, Ar-H), 11.20 (*s*, 1H, NH of isatin), 12.03 (*s*, 1H, hydrazide NH), 13.45 (*s*, 1H, Indole NH); IR (KBr, *ν* cm^−1^) 3528, 3448, 3176 (3NH) and 1711, 1687 (2 C=O); Calcd. Anal. for C_19_H_16_N_4_O_2_: C, 68.66; H, 4.85; N, 16.86; found C, 68.14; H, 5.37; N, 16.68.

##### 4.1.2.11. N'-(1-Methyl-2-oxoindolin-3-ylidene)-1H-indole-3-carbohydrazide 9a

Yellow powder; m.p. 273–275 °C; (yield 66%); ^1^H NMR *δ ppm*: 3.22, 3.26 (*s*, 3H, *N*-CH_3_), 7.10–7.18 (*m*, 2H, Ar-H), 7.22–7.30 (*m*, 2H, Ar-H), 7.43–7.49 (*m*, 1H, Ar-H), 7.53–7.58 (*m*, 1H, Ar-H), 7.66 (d, 0.3H, Ar-H, *J* = 8.0 Hz), 8.17 (d, 0.7H, Ar-H, *J* = 8.0 Hz), 8.24–8.33 (*m*, 1.3H, Ar-H), 8.67 (*s*, 0.7H, Ar-H), 11.24, 13.38 (*s*, 1H, hydrazide NH), 12.04, 12.07 (*s*, 1H, Indole NH); ^13 ^C NMR *δ ppm*: 26.65 (*N*-CH_3_), 108.60, 110.78, 113.46, 120.48, 121.10, 121.34, 122.45, 123.69, 124.03, 126.55, 131.90, 132.33, 137.24, 144.14 (Aromatic carbons), 161.95 (C=O hydrazide), 162.55 (C=O isatin)); IR (KBr, *ν* cm^−1^) 3436, 3117 (2NH) and 1774, 1725 (2 C=O); Calcd. Anal. for C_18_H_14_N_4_O_2_: C, 67.92; H, 4.43; N, 17.60; found C, 67.65; H, 4.97; N, 17.54.

##### 4.1.2.12. N'-(2-Oxo-1-propylindolin-3-ylidene)-1H-indole-3-carbohydrazide 9b

Yellow powder; m.p. 248–250 °C; (yield 69%); ^1^H NMR *δ ppm*: 0.92 (*t*, 3H, –CH_2_CH_3_, *J* = 8.0 Hz), 1.65–1.74 (*m*, 2H, –CH_2_CH_3_), 3.76 (*t*, 2H, *N*-CH_2_, *J* = 8.0 Hz), 7.17 (*t*, 1H, Ar-H, *J* = 8.0 Hz), 7.23–7.30 (*m*, 3H, Ar-H), 7.44 (*t*, 1H, Ar-H, *J* = 8.0 Hz), 7.55–7.58 (*m*, 1H, Ar-H), 7.68 (d, 1H, Ar-H, *J* = 8.0 Hz), 8.23–8.25 (*m*, 1H, Ar-H), 8.29 (*s*, 1H, Ar-H), 12.06 (*s*, 1H, hydrazide NH), 13.40 (*s*, 1H, Indole NH); ^13 ^C NMR *δ ppm*: 11.67 (–CH_2_CH_3_), 20.95 (–CH_2_CH_3_), 41.24 (*N*-CH_2_), 108.10, 110.53, 113.00, 120.08, 120.78, 120.94, 122.01, 123.23, 123.45, 126.19, 131.42, 131.84, 134.42, 136.80, 143.00 (Aromatic carbons), 161.51 (C=O hydrazide), 162.10 (C=O isatin)); IR (KBr, *ν* cm^−1^) 3310, 3151 (2NH) and 1727, 1663 (2 C=O); Calcd. Anal. for C_20_H_18_N_4_O_2_: C, 69.35; H, 5.24; N, 16.17; found C, 69.20; H, 5.73; N, 16.19.

##### 4.1.2.13. N'-(1-Benzyl-2-oxoindolin-3-ylidene)-1H-indole-3-carbohydrazide 9c

Yellow powder; m.p. 274–276 °C; (yield 72%); ^1^H NMR *δ ppm*: 5.00 (*s*, 2H, CH_2_), 7.06 (d, 1H, Ar-H, *J* = 8.0 Hz), 7.16 (*t*, 1H, Ar-H, *J* = 8.0 Hz), 7.24–7.31 (*m*, 3H, Ar-H), 7.34–7.43 (*m*, 5H, Ar-H), 7.57 (d, 1H, Ar-H, *J* = 8.0 Hz), 7.70 (d, 1H, Ar-H, *J* = 8.0 Hz), 8.25 (d, 1H, Ar-H, *J* = 8.0 Hz), 8.32 (*s*, 1H, Ar-H), 12.09 (*s*, 1H, hydrazide NH), 13.35 (*s*, 1H, Indole NH); ^13 ^C NMR *δ ppm*: 43.03 (CH_2_), 108.09, 110.81, 113.03, 120.21, 120.86, 120.95, 122.06, 123.28, 123.70, 126.18, 127.83, 128.10, 129.22, 131.33, 131.89, 134.29, 136.20, 136.80, 142.62 (Aromatic carbons), 161.52 (C=O hydrazide), 162.13 (C=O isatin); IR (KBr, *ν* cm^−1^) 3450, 3210 (2NH) and 1748, 1688 (2 C=O); Calcd. Anal. for C_24_H_18_N_4_O_2_: C, 73.08; H, 4.60; N, 14.20; found C, 72.58; H, 5.14; N, 14.06.

##### 4.1.2.14. N'-(5-Chloro-2-oxo-1-propylindolin-3-ylidene)-1H-indole-3-carbohydrazide 9d

Yellow powder; m.p. 262–263 °C; (yield 70%); IR (KBr, *ν* cm^−1^) 3450, 3221 (2NH) and 1730, 1660 (2 C=O); ^1^H NMR *δ ppm*: 0.90 (*t*, 3H, –CH_2_CH_3_, *J* = 8.0 Hz), 1.62–1.71 (*m*, 2H, –CH_2_CH_3_), 3.72 (*t*, 2H, *N*-CH_2_, *J* = 8.0 Hz), 7.22–7.28 (*m*, 3H, Ar-H), 7.45 (d, 1H, Ar-H, *J* = 8.0 Hz), 7.55 (d, 1H, Ar-H, *J* = 8.0 Hz), 7.65 (*s*, 1H, Ar-H), 8.22 (d, 1H, Ar-H, *J* = 8.0 Hz), 8.34 (*s*, 1H, Ar-H), 12.06 (*s*, 1H, hydrazide NH), 13.26 (*s*, 1H, Indole NH); ^13 ^C NMR *δ ppm*: 11.61 (–CH_2_CH_3_), 20.89 (–CH_2_CH_3_), 41.37 (*N*-CH_2_), 107.71, 112.06, 112.55, 112.99, 120.35, 120.99, 121.81, 122.06, 123.26, 126.35, 127.70, 130.66, 133.15, 136.73, 141.63 (Aromatic carbons), 161.28 (C=O hydrazide), 162.20 (C=O isatin); IR (KBr, *ν* cm^−1^) 3450, 3210 (2NH) and 1748, 1688 (2 C=O); Calcd. Anal. for C_20_H_17_ClN_4_O_2_: C, 63.08; H, 4.50; N, 14.71; found C, 62.89; H, 4.94; N, 14.71.

##### 4.1.2.15. N'-(5-Bromo-2-oxo-1-propylindolin-3-ylidene)-1H-indole-3-carbohydrazide 9e

Orange powder; m.p. 255–256 °C; (yield 75%); ^1^H NMR *δ ppm*: 0.95 (*t*, 3H, –CH_2_CH_3_, *J* = 8.0 Hz), 1.70–1.79 (*m*, 2H, –CH_2_CH_3_), 3.78 (*t*, 2H, *N*-CH_2_, *J* = 8.0 Hz), 6.94 (d, 1H, Ar-H, *J* = 8.0 Hz), 7.21–7.30 (*m*, 2H, Ar-H), 7.51 (d, 1H, Ar-H, *J* = 8.0 Hz), 7.55 (d, 1H, Ar-H, *J* = 8.0 Hz), 7.75 (*s*, 1H, Ar-H), 8.24 (d, 1H, Ar-H, *J* = 8.0 Hz), 8.35 (*s*, 1H, Ar-H), 12.10 (*s*, 1H, hydrazide NH), 13.29 (*s*, 1H, Indole NH); ^13 ^C NMR *δ ppm*: 11.62 (–CH_2_CH_3_), 20.89 (–CH_2_CH_3_), 41.35 (*N*-CH_2_), 107.68, 112.51, 112.99, 115.33, 121.00, 122.05, 122.17, 123.04, 123.25, 126.36, 132.37, 132.98, 133.45, 136.72, 142.00 (Aromatic carbons), 161.13 (C=O hydrazide), 162.22 (C=O isatin); IR (KBr, *ν* cm^−1^) 3450, 3179 (2NH) and 1745, 1688 (2 C=O); Calcd. Anal. for C_20_H_17_BrN_4_O_2_: C, 56.48; H, 4.03; N, 13.17; found C, 56.06; H, 4.43; N, 13.16.

### 4.2. Biological evaluation

#### 4.2.1. Cell culture

HT29 and SW620 colorectal cancer cell lines and HFF-1 fibroblast (ATCC, Rockville, USA) were used. HT29 cells were cultured and maintained in Dulbecco’s Modified Eagle’s Medium (DMEM) (GIBCO, by Thermo Fischer Scientific, NY, USA) supplemented with 10% foetal bovine serum (FBS), 100 units/mL penicillin, and 100 µg streptomycin. SW620 and HFF-1 cells were cultured in Roswell Park Memorial Institute medium (RPMI-1640) (GIBCO, by Thermo scientific, NY, USA) supplemented with 10% FBS and 1% penicillin and streptomycin (Napolitano et al., 2015). All cultures were incubated at 37 °C and humidified atmosphere of 5% CO_2_.

#### 4.2.2. Cell viability assay

The cytotoxicity effect of compounds on the colorectal cancer cell lines, HT29 and SW620 in addition to the normal human fibroblasts was measured by MTT (3–(4, 5-dimethylthiazol-2-yl)-2,5-diphenyltetrazolium bromide) (Sigma-Aldrich, St. Louis, MO, USA) as previously described^39^. Briefly, cells were seeded in 96 well culture plates at 5000/well for HT29 and 10,000/well for SW620 for 24 h. Cells were then incubated with different compounds from WAG1 series (1–15) 24 h at 37 °C and humidified 5% CO_2_ incubator. Freshly prepared 10 µl of 3–(4,5-dimethylthiazol-2-yl)-2,5-diphenyltetrazolium bromide (MTT 5 mM) solution were added to the cells and further incubated for 2 h. Thereafter, 100 µl of dimethyl sulphoxide (DMSO) were added in each well and the crystals were dissolved through careful pipetting. In certain experiment, cells were treated different concentration of 5FU for 72 h. The absorbance of the product was measured at 540 nm using a Synergy™ 2 multi-mode microplate reader (Biotech, VA, USA). The experiments were performed in triplicate for each condition.

#### 4.2.3. Measurement of apoptosis by annexin V-FITC/PI assay:

Induction of apoptosis was measured by Dead Cell Apoptosis Kit with Annexin V FITC and PI, for flow cytometry (Thermofischer scientific, OR, USA) according to the manufacturer’s instruction. As described previously (39), cells were seeded in a 6-well plate (3 × 10^5^ cells per well) and treated with the various compound for 24 h. Both floating and adherent cells were harvested, pooled together, and incubated with Annexin V-FITC and PI for 15 min on ice in dark. The cells were analysed by BD FACSCalibur™ cell analyser (BD Biosciences, CA, USA) at an emission of 530 nm (FL1 channel) and >575 nm (FL3).

#### 4.2.4. Western blot analysis:

All cells were seeded in a 100 mm dish (1 × 10^6^ cells per dish) in 5% CO_2_ at 37 °C in the appropriate culture medium. The cells with around 50% confluency were treated with compounds for 24 h. At the experiment day, cells were washed with 1x PBS, harvested and lysed in RIPA lysis buffer, combined with protease inhibitors, (Sigma-Aldrich, St. Louis, MO, USA) as described previously^39^). The total protein concentration was evaluated by the colorimetric Bradford protein assay (BIO-RAD inc, CA, USA) at 595 nm absorbance. Lysates were loaded in equal concentration and separated by sodium dodecyl sulfate-polyacrylamide gel electrophoresis (SDS-PAGE) and then transferred to a nitrocellulose membrane by semi-dry. Blocking of the membrane was done by 5% non-fat dried milk for one hour, incubated with the primary antibodies. The primary antibodies used were Bcl2 (cat. no. sc-7382), BclxL (cat. no. sc-8392) and β Actin (cat. no. sc-69879) from (Santa Cruz Biotechnology, Inc., Dallas, TX, USA). The secondary antibodies used were goat anti-mouse IgG-HRP (cat. no. sc-2005) and mouse anti-rabbit IgG-HRP (cat. no. sc-2357) from (Santa Cruz Biotechnology, Inc., Dallas, TX, USA). Detection was done with Luminol HRP chemiluminescence substrate (cat. no. sc-2048) from (Santa Cruz Biotechnology, Inc., Dallas, TX, USA) and then visualised by c-digit blot-scanner (LI-COR, Nebraska, USA).

#### 4.2.5. Statistical analysis

The statistical analysis by the one-way ANOVA test was performed by GraphPad prism. Results were considered significant if the *p-Values* were <0.05.

## Supplementary Material

Supplemental Material
