# Supplementary figures and images for: Development of 2-oindolin-3-ylidene-indole-3-carbohydrazide derivatives as novel apoptotic and anti-proliferative agents towards colorectal cancer cells
Source: J Enzyme Inhib Med Chem. 2020 Dec 20;36(1):319–28. doi: 10.1080/14756366.2020.1862100 (PMC7751403; doi:10.1080/14756366.2020.1862100)

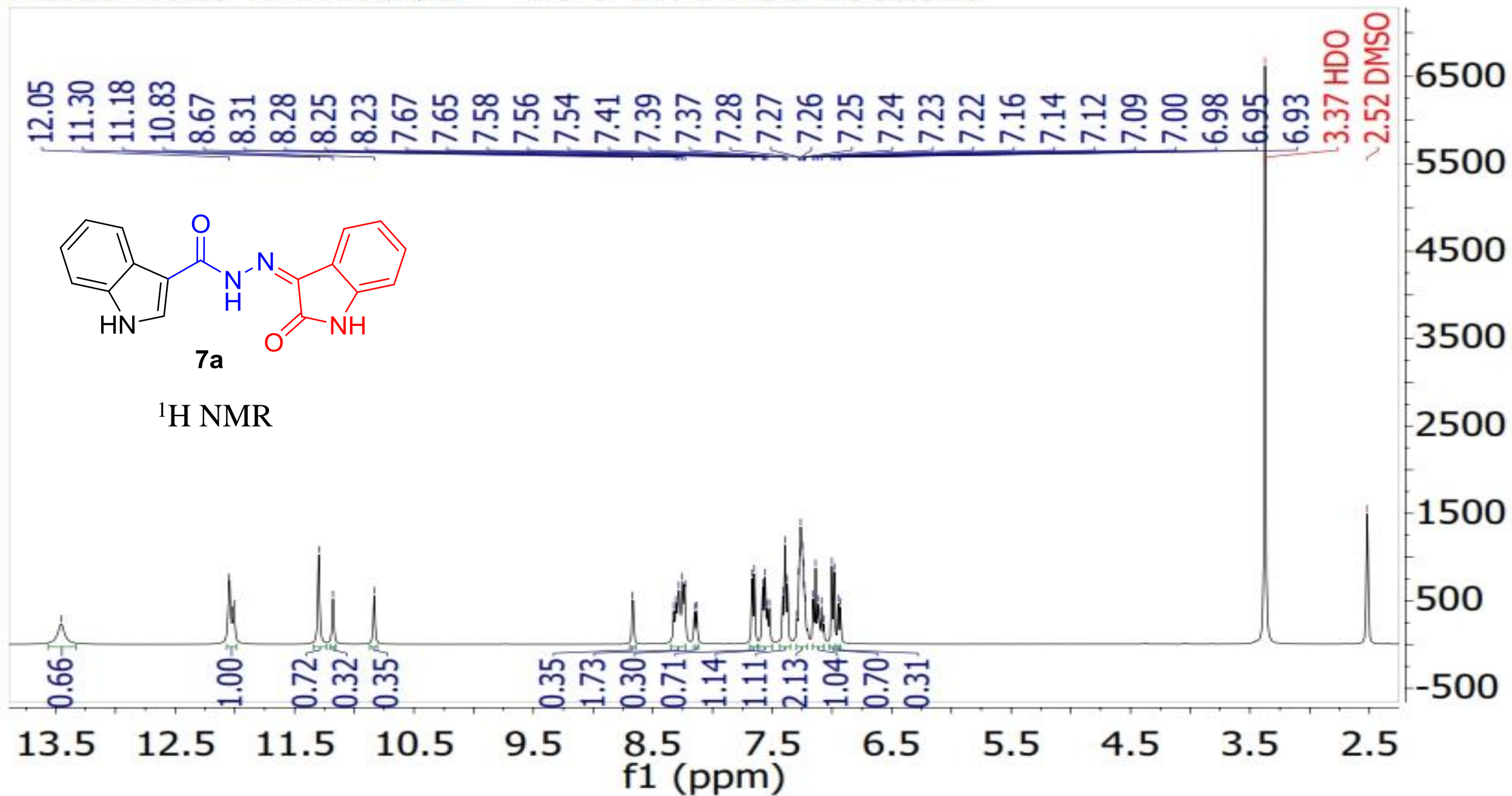

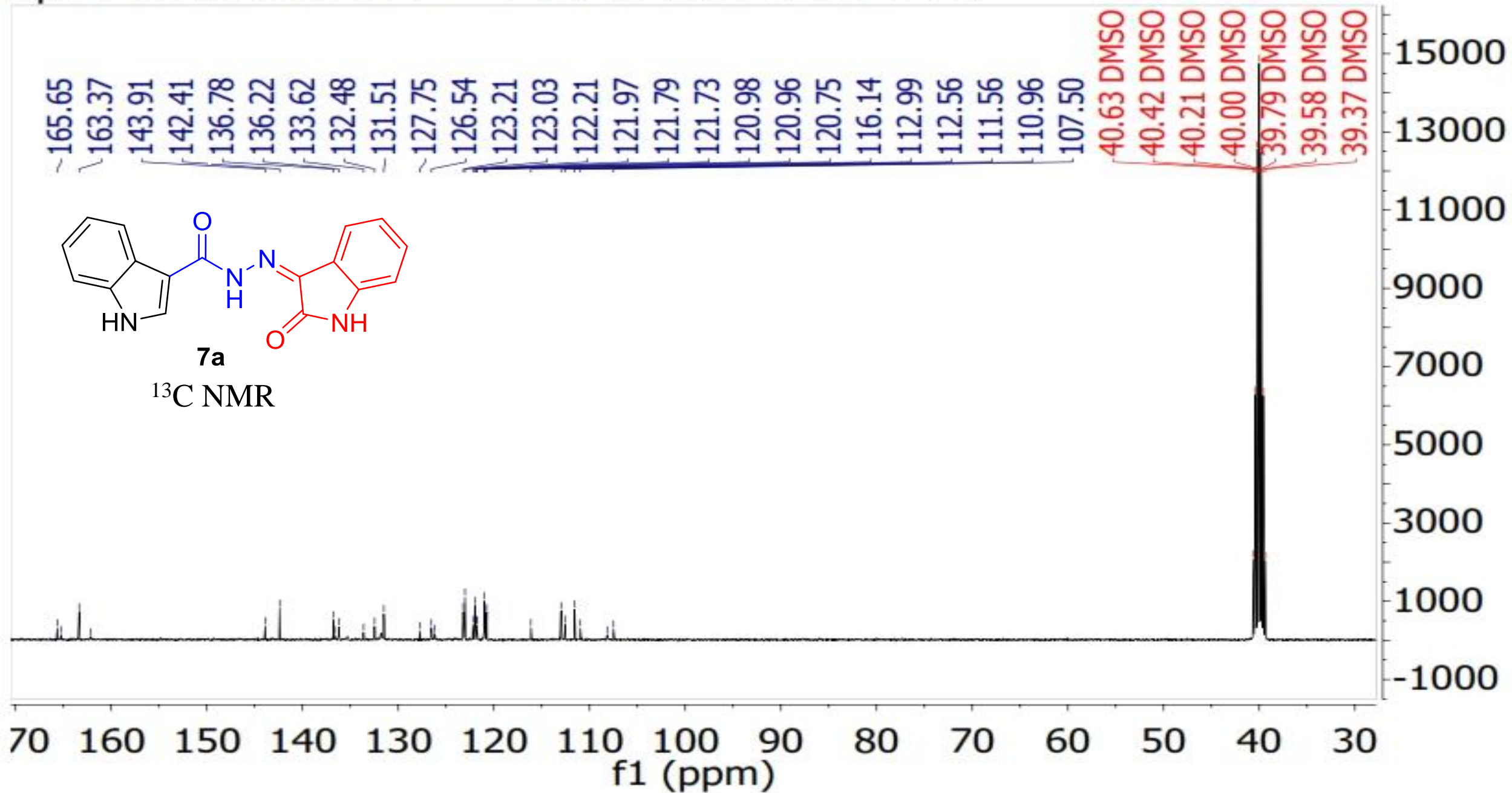

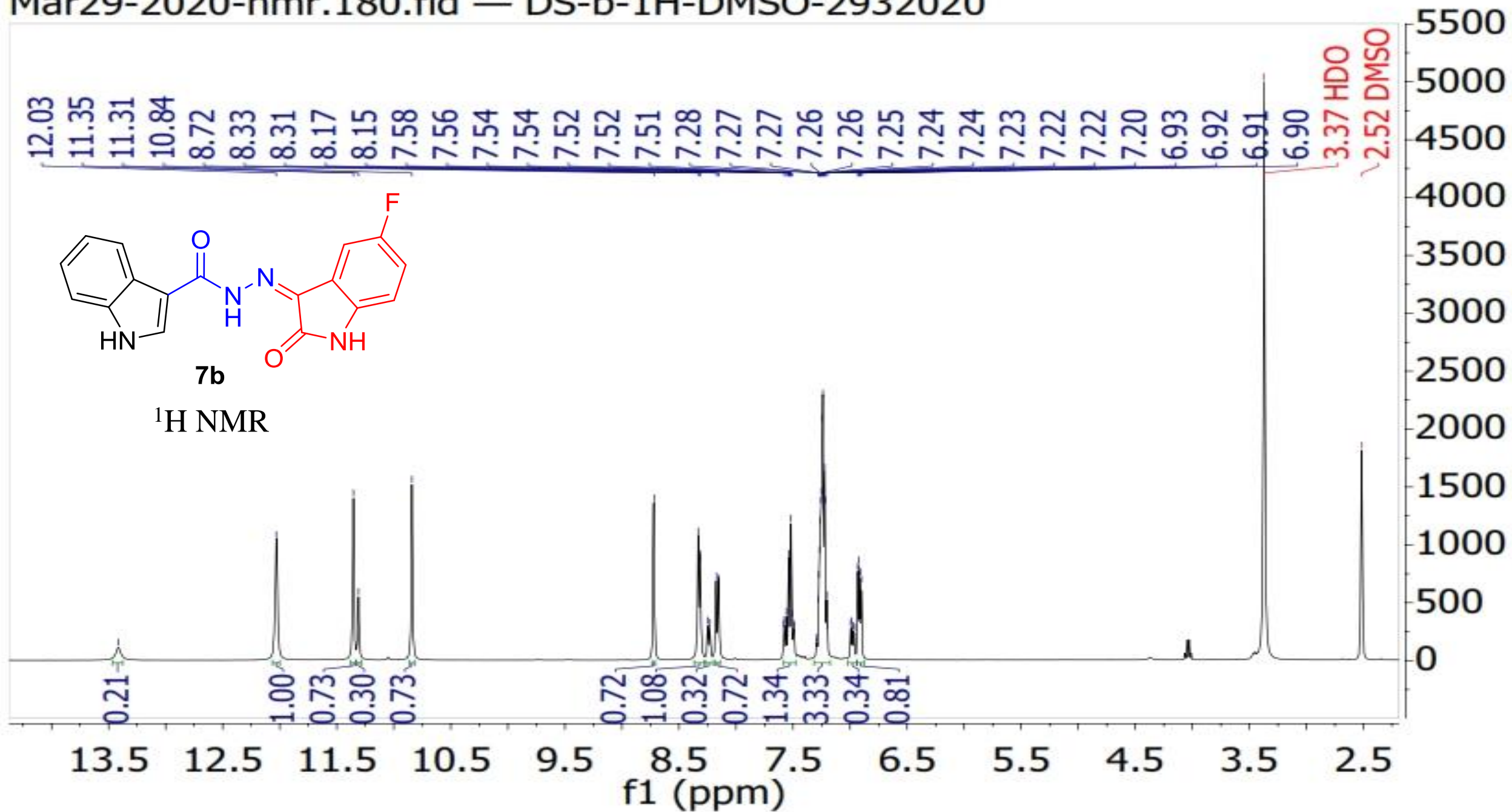

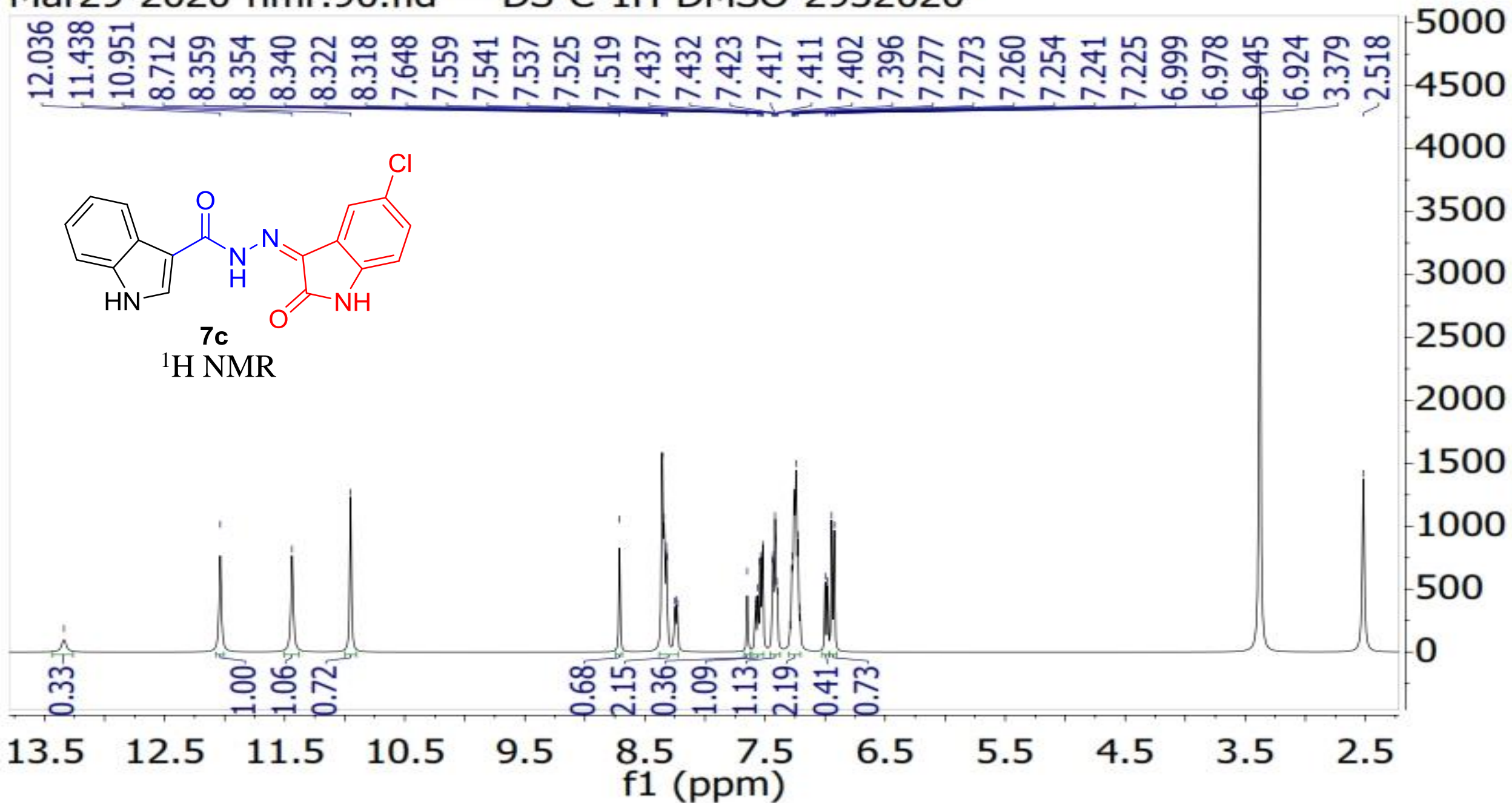

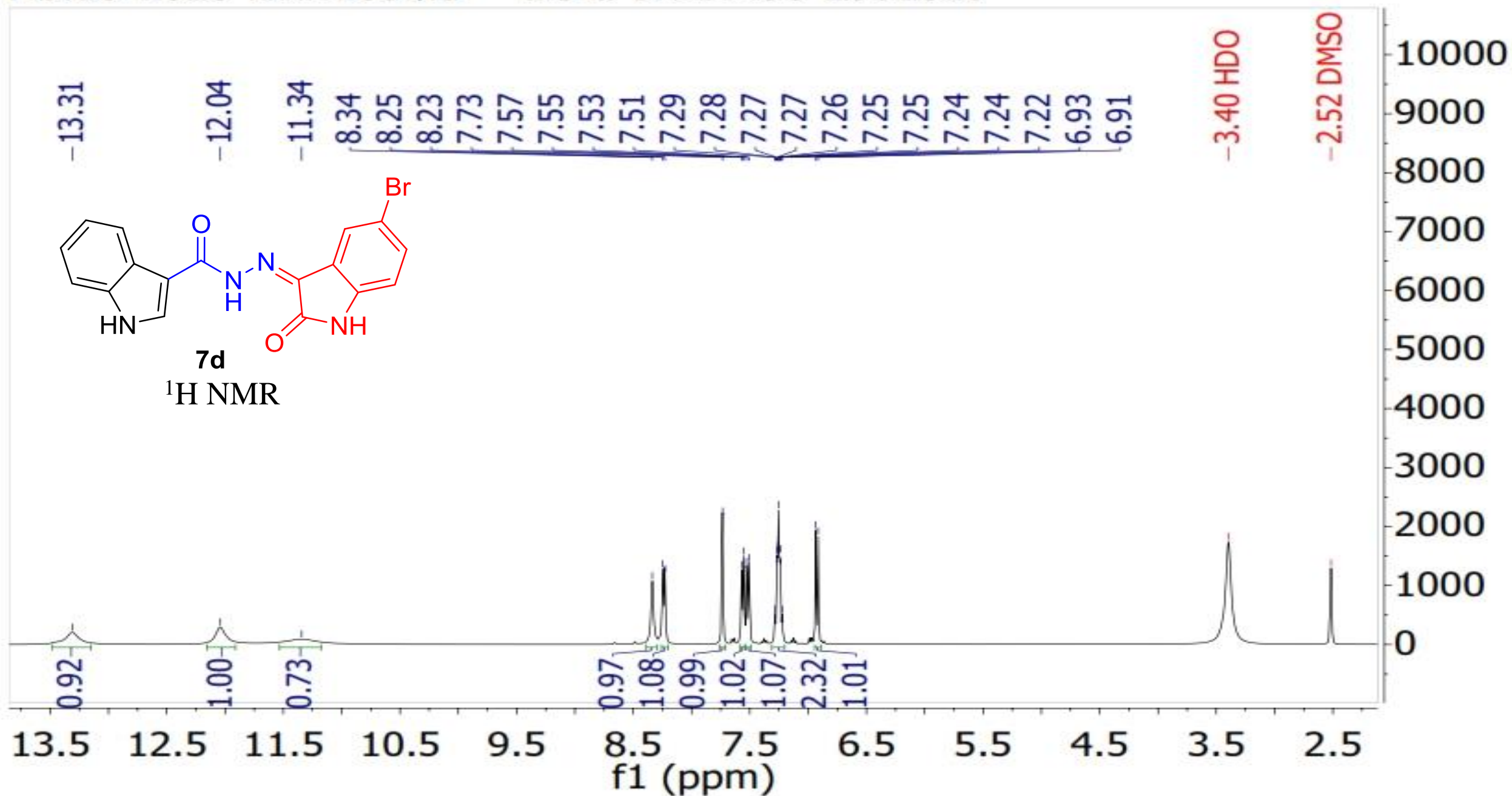

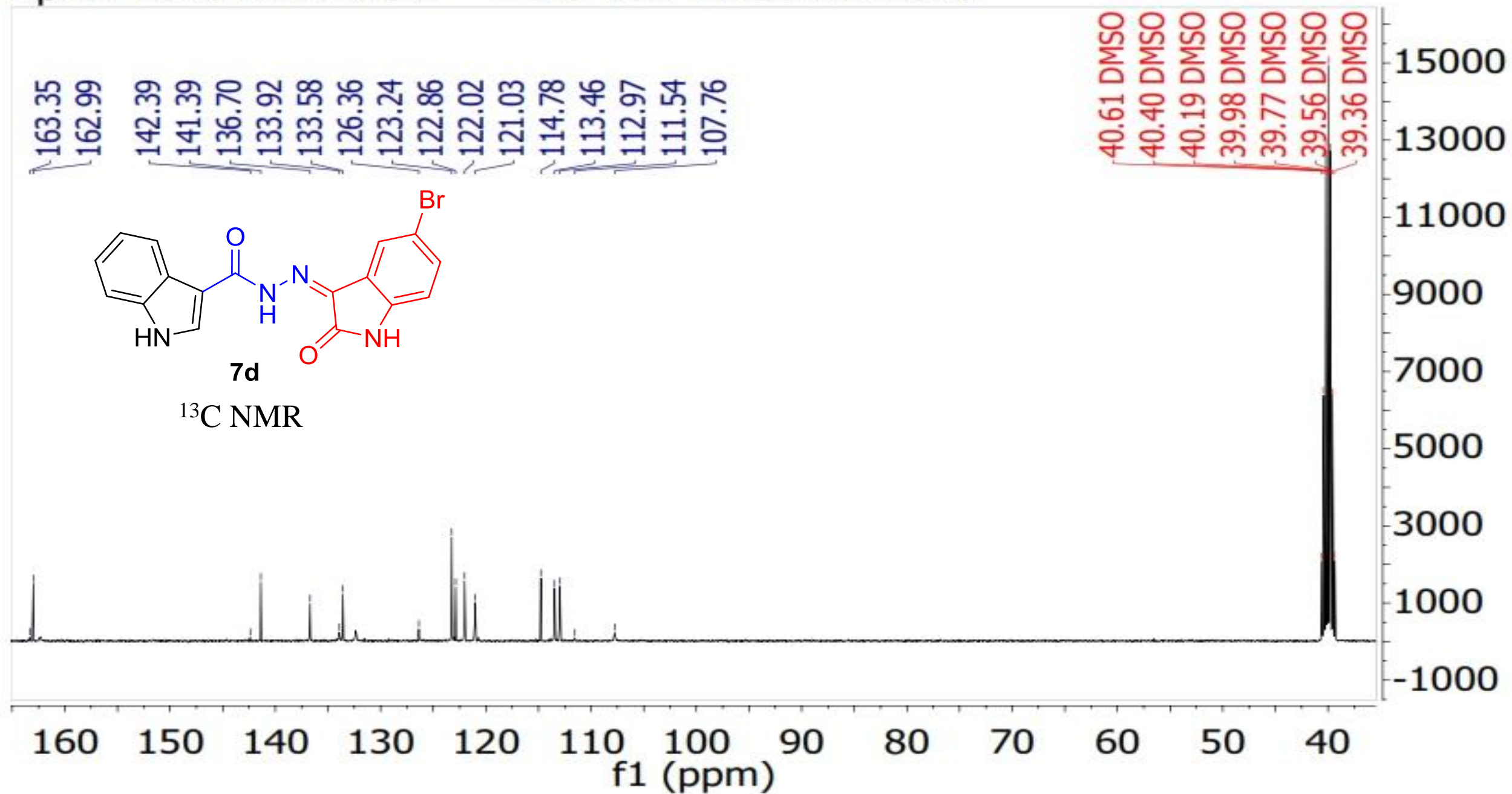

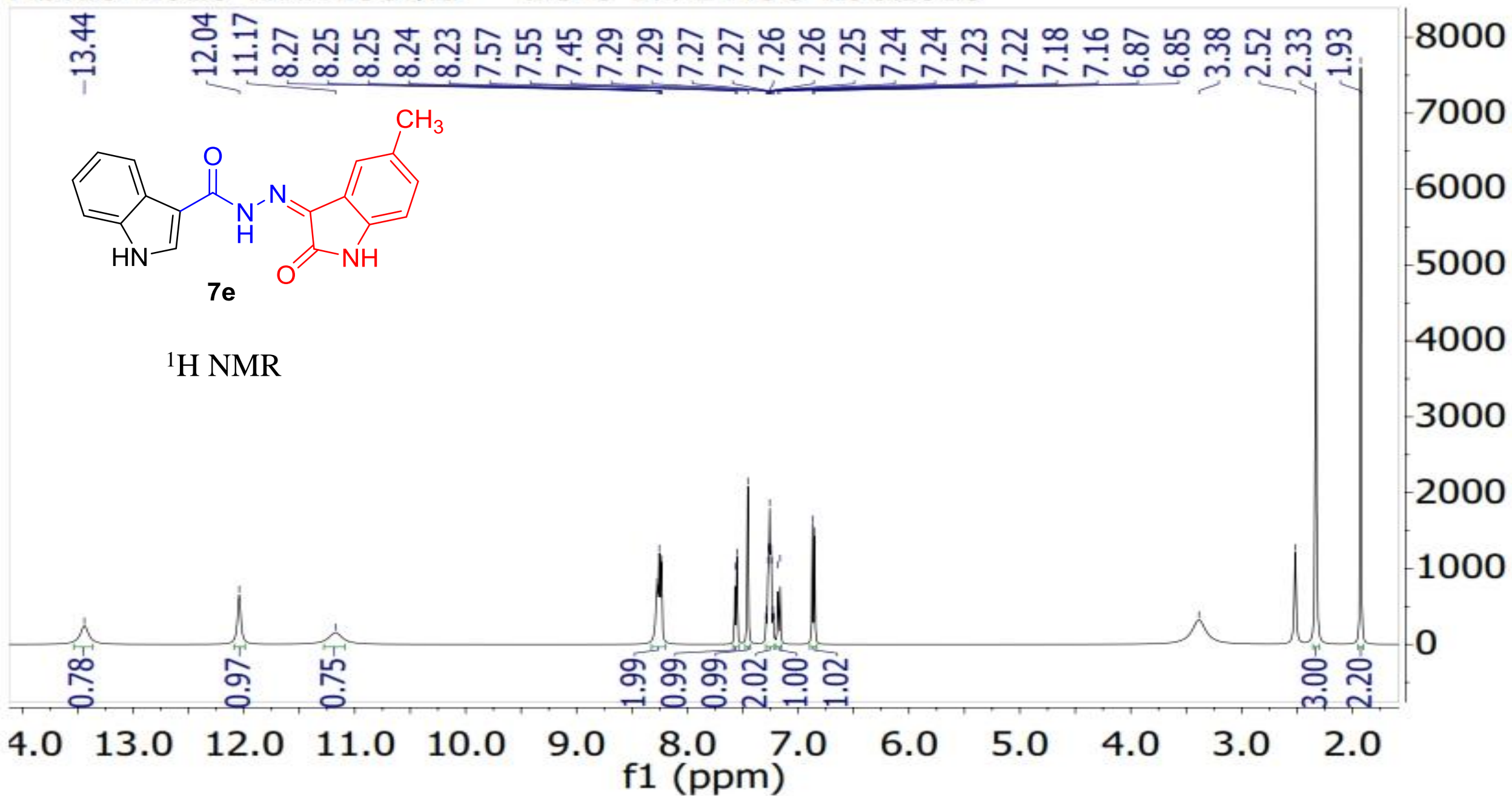

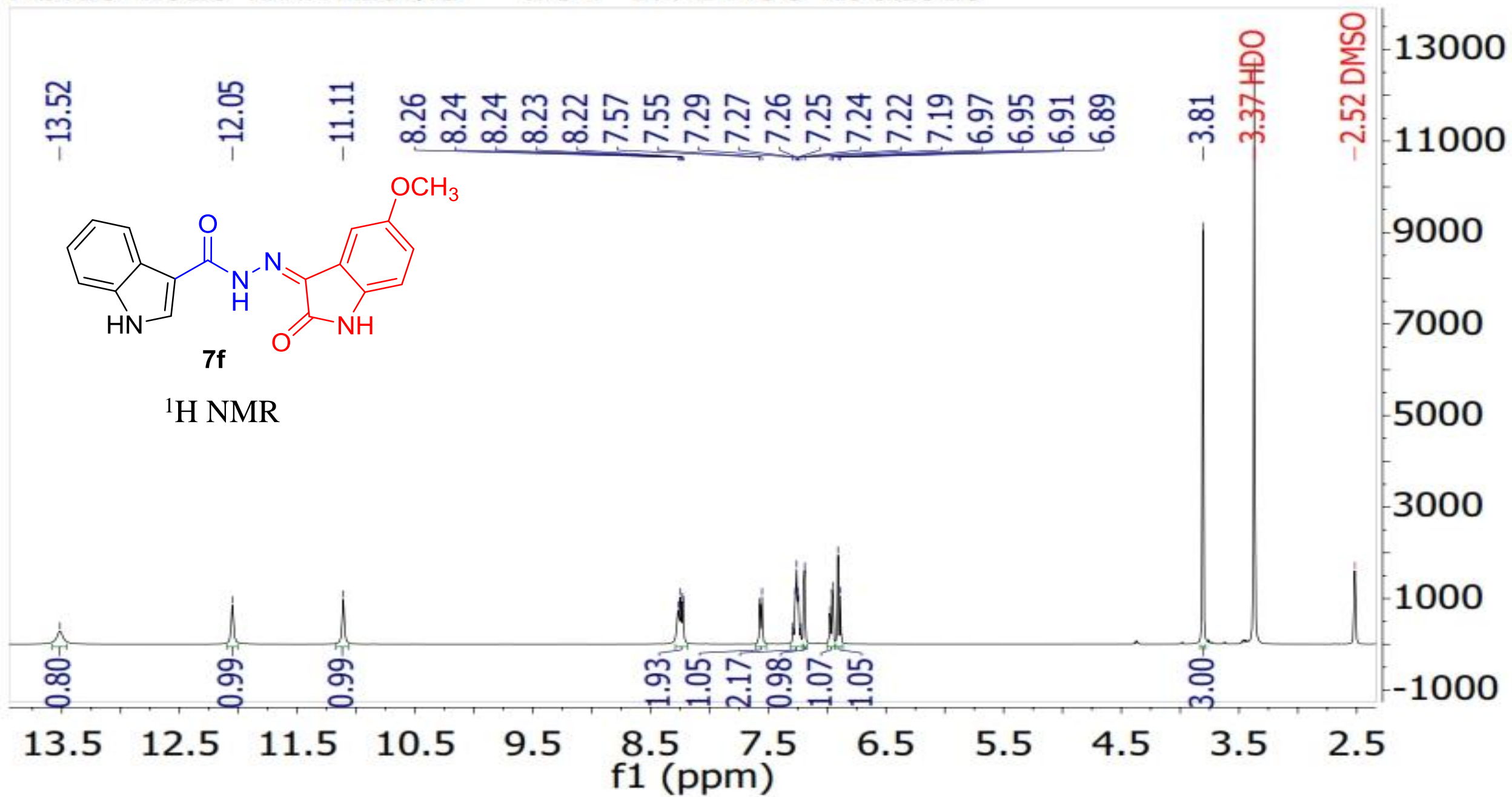

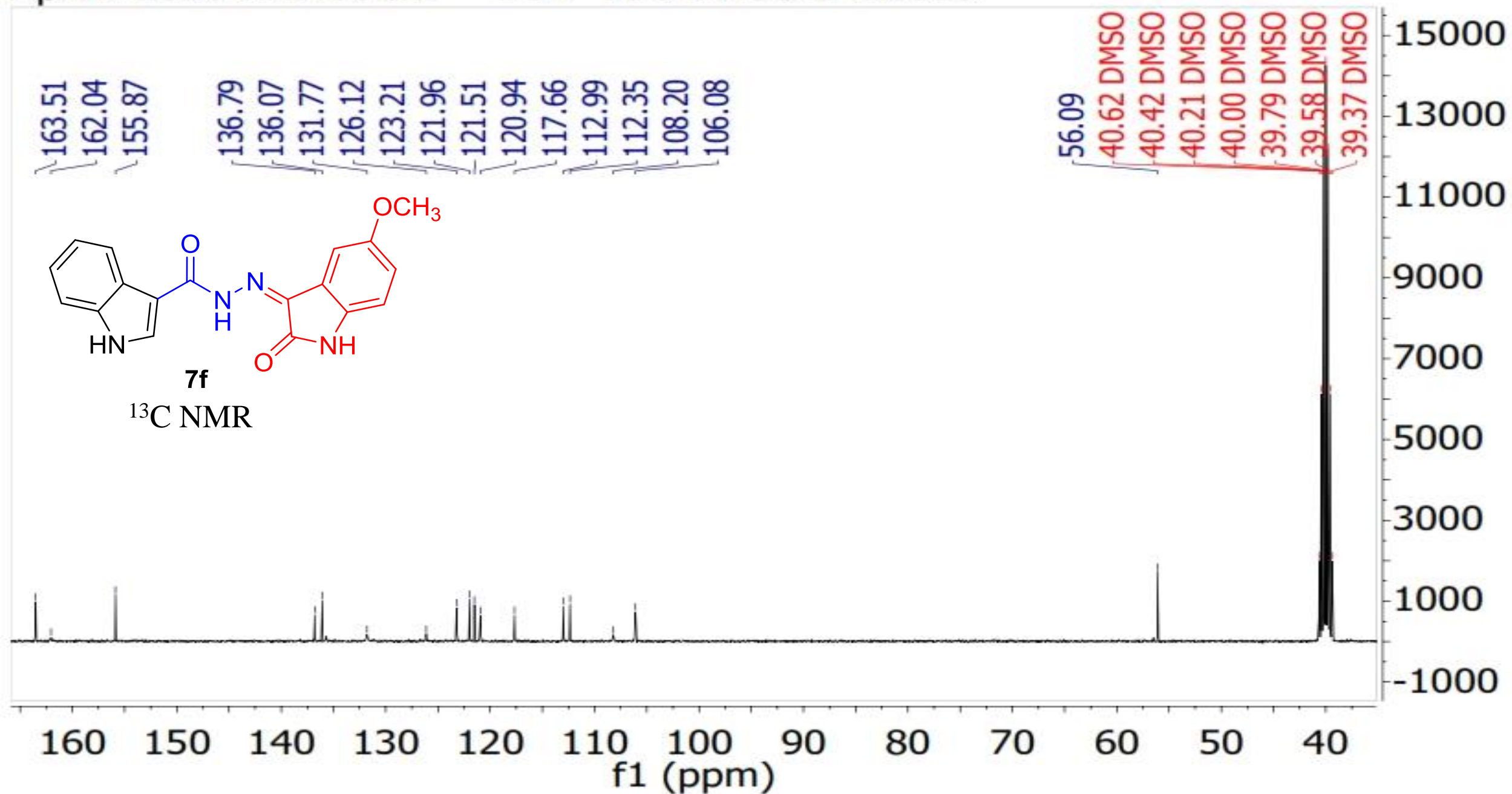

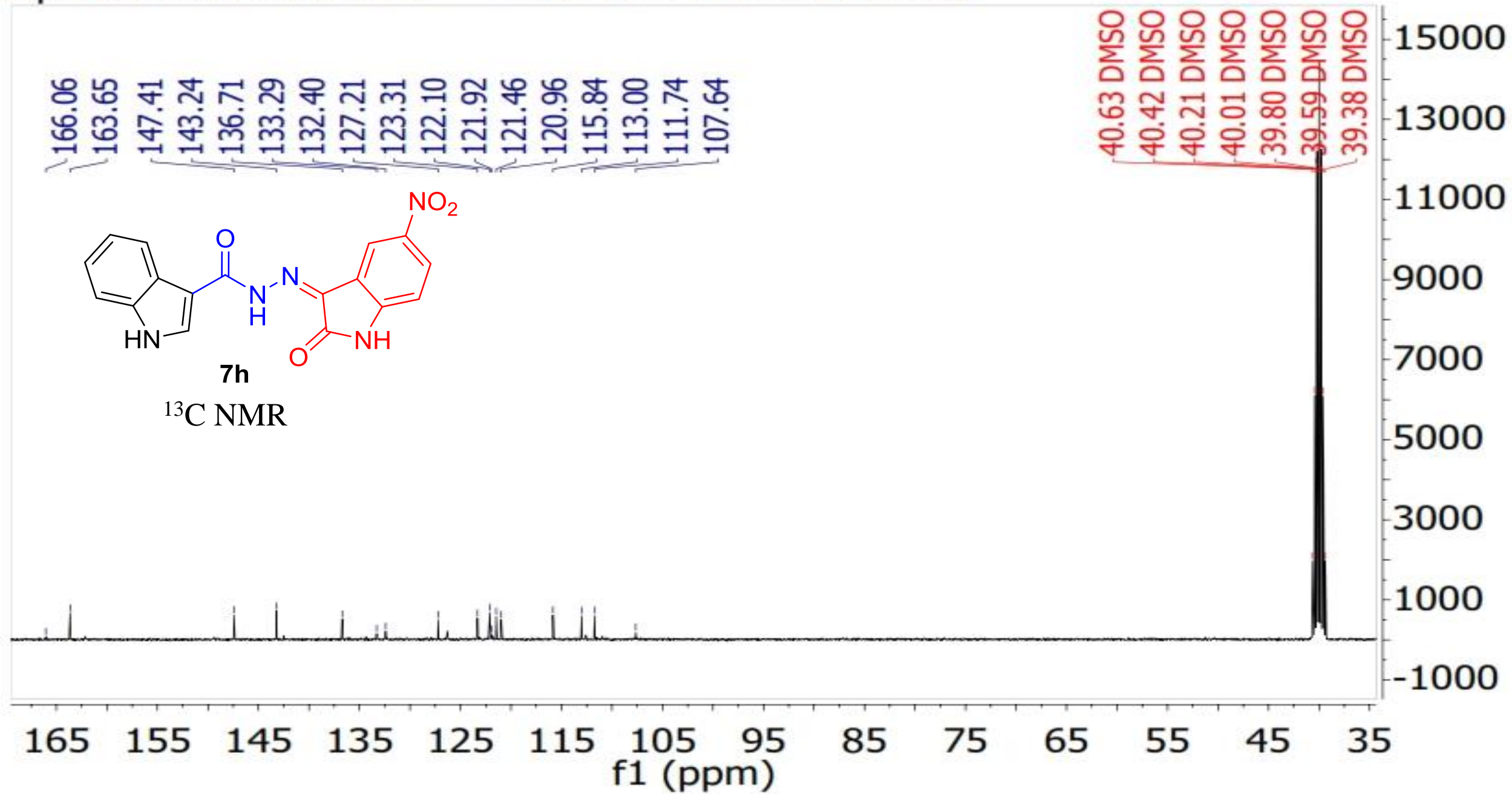

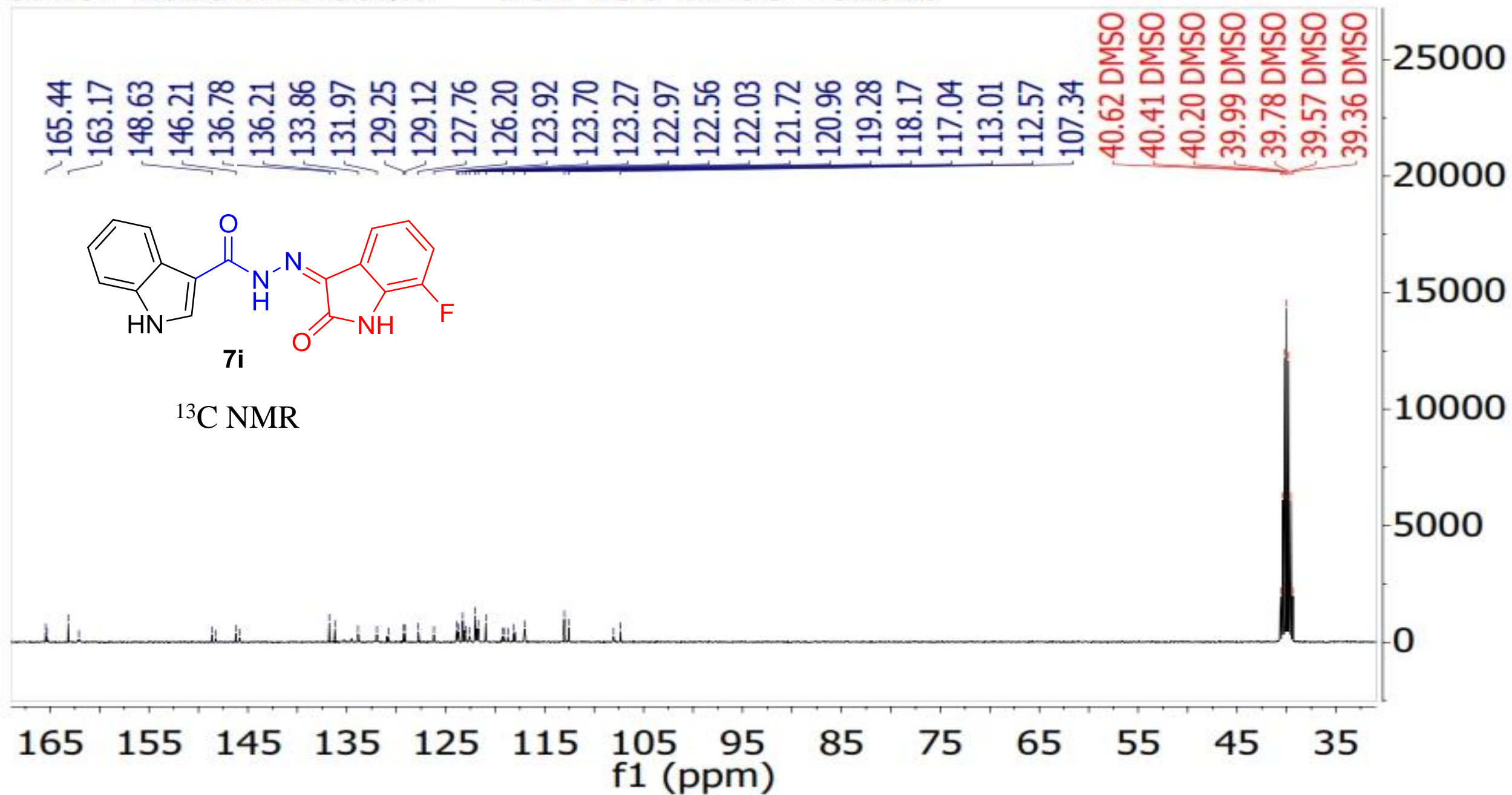

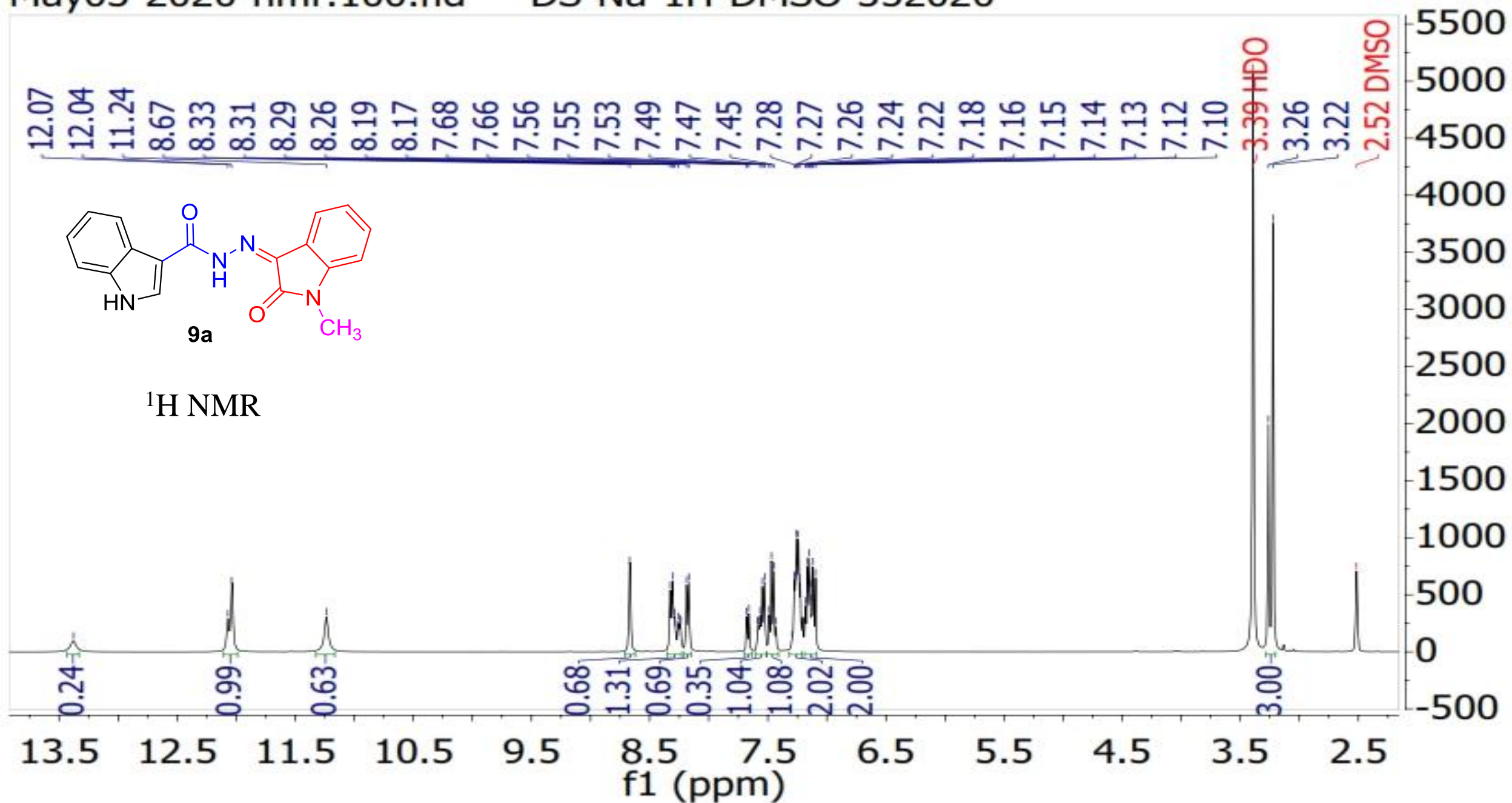

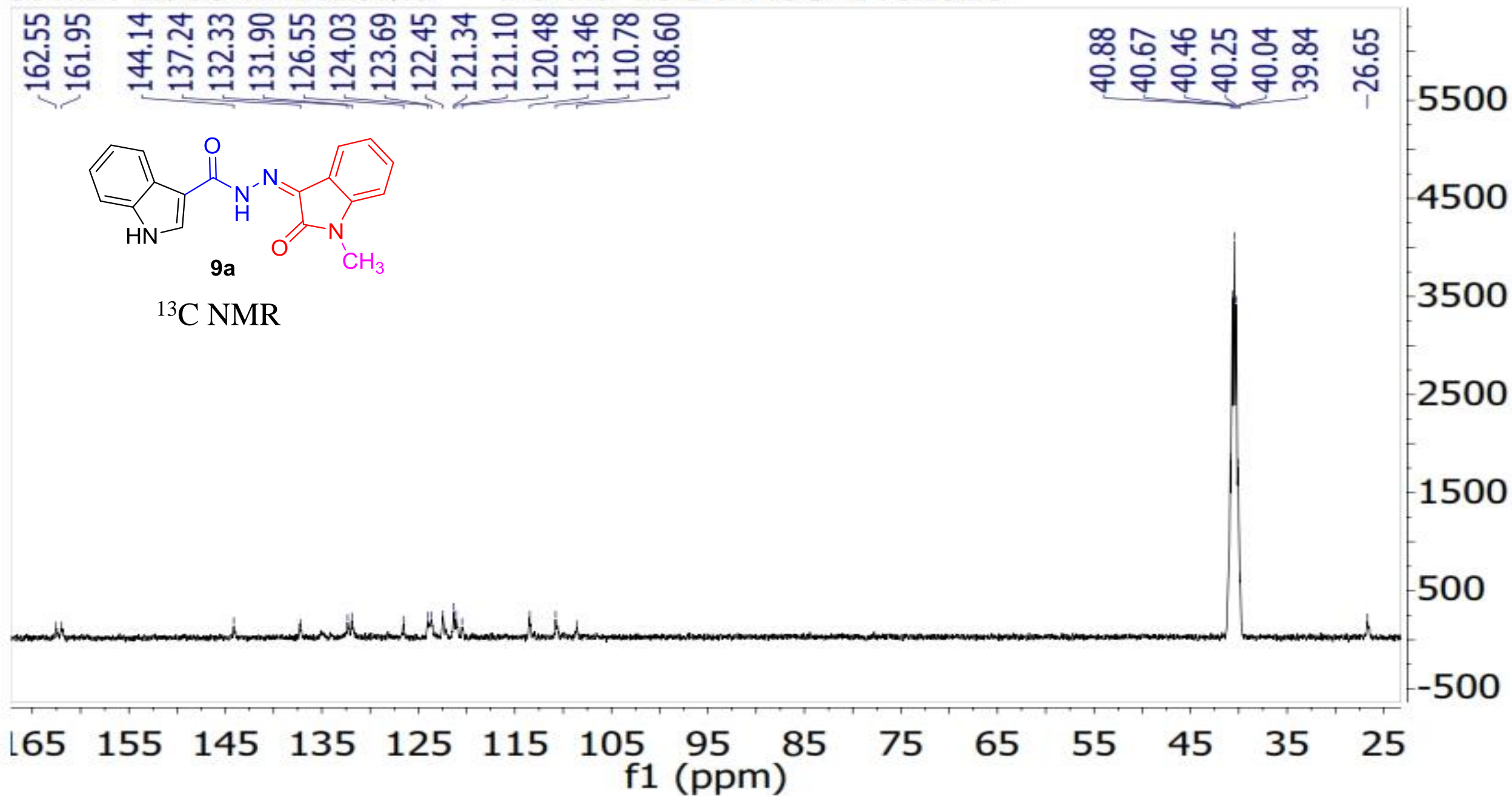

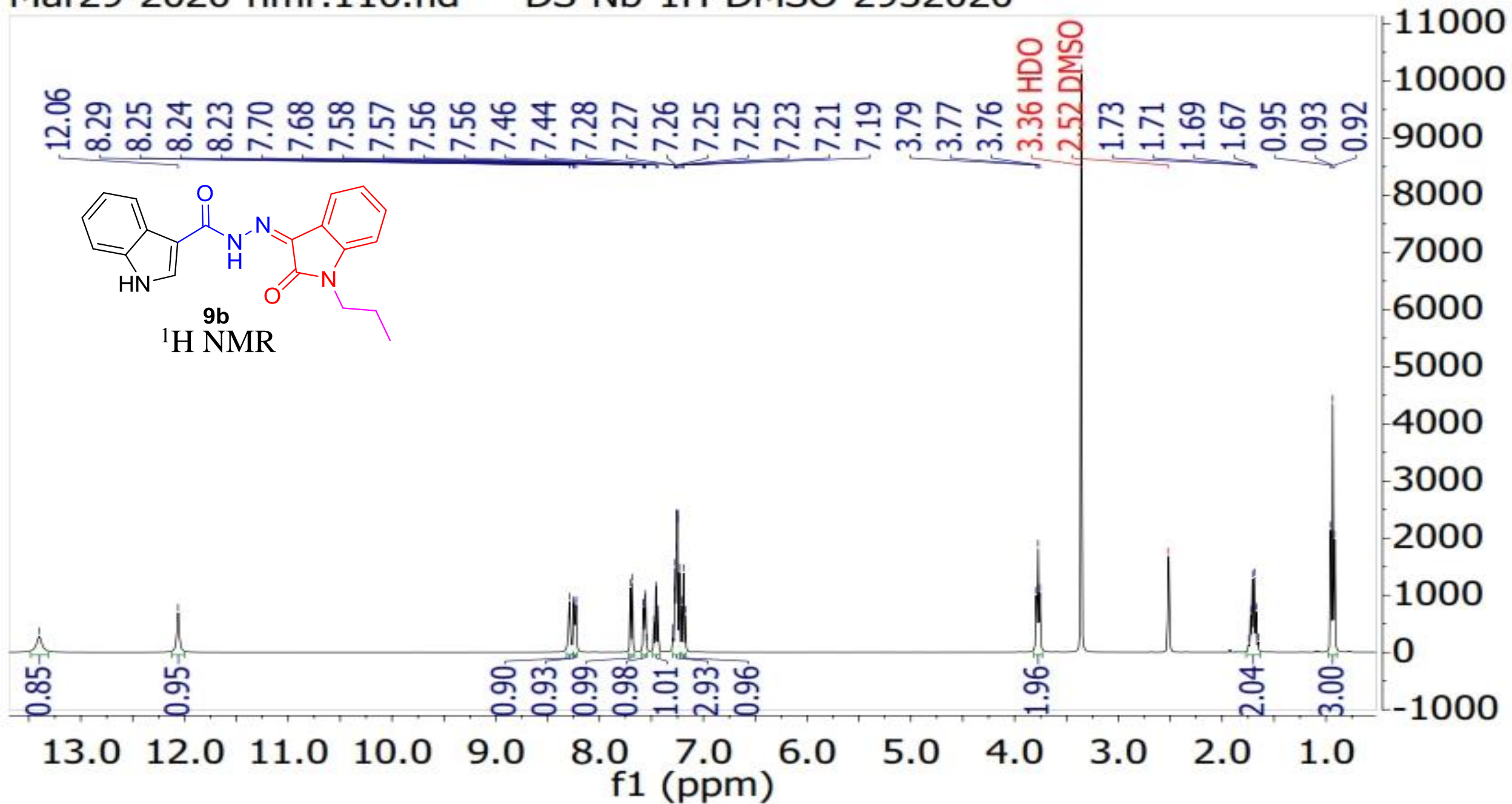

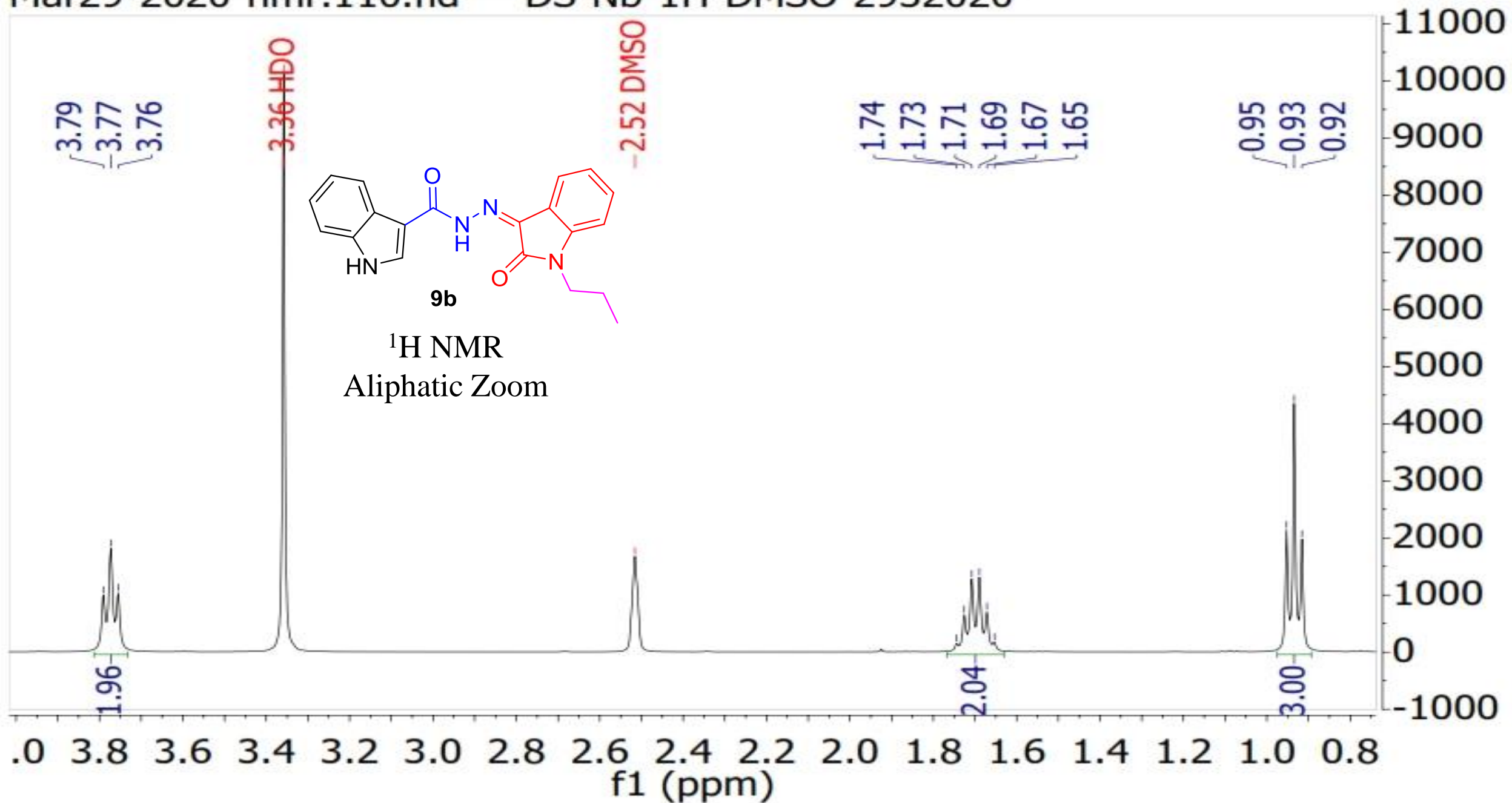

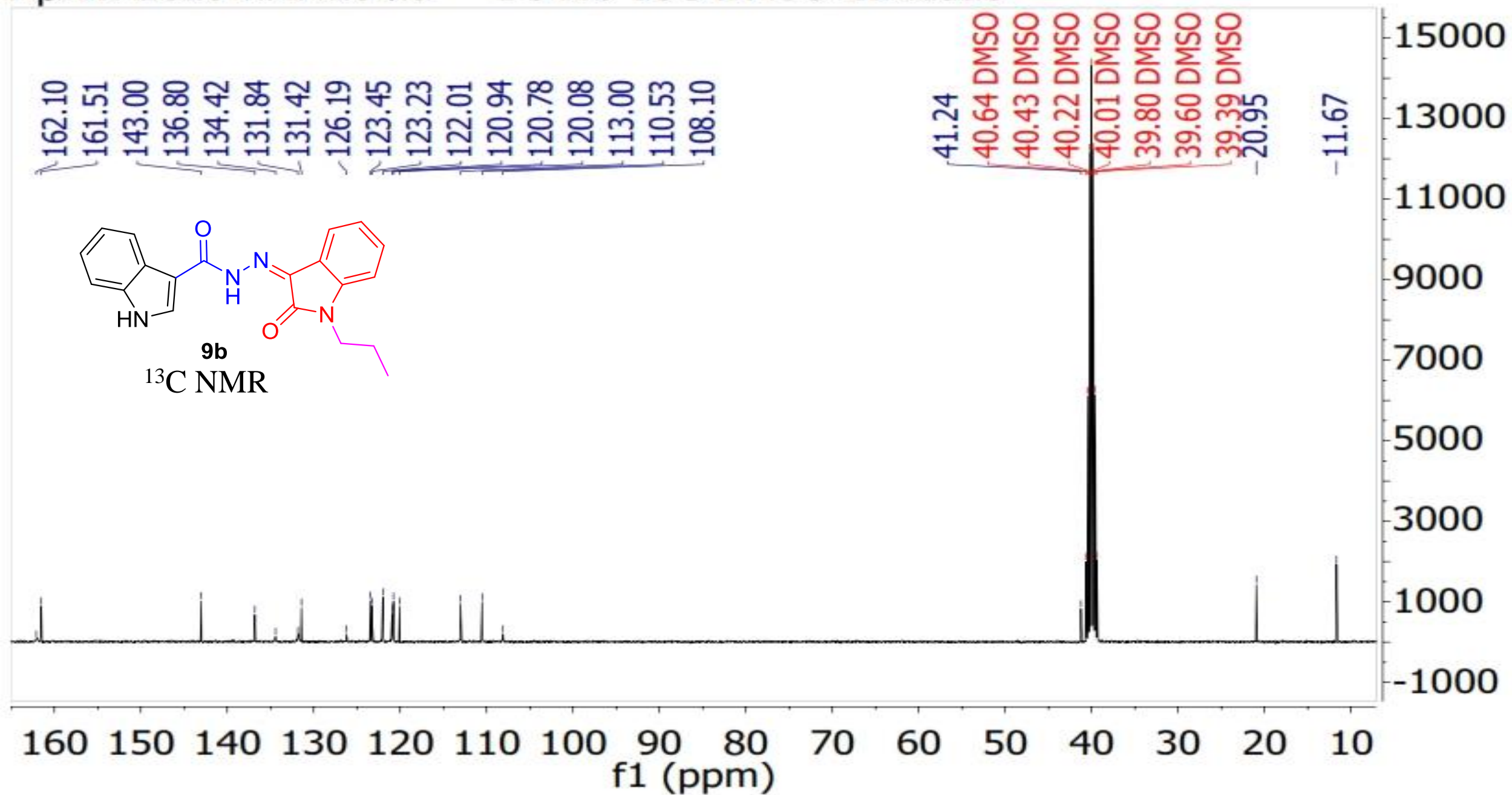

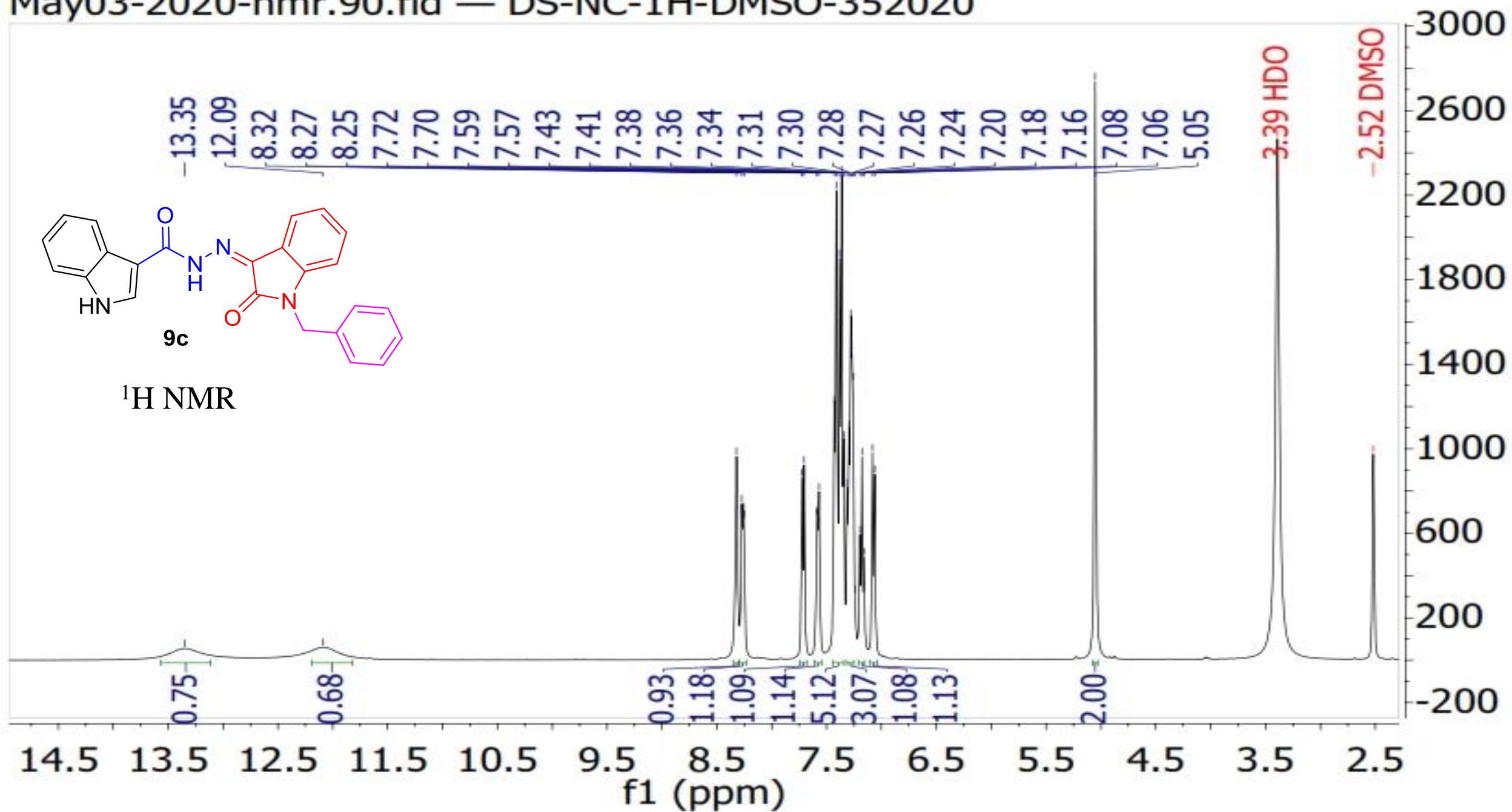

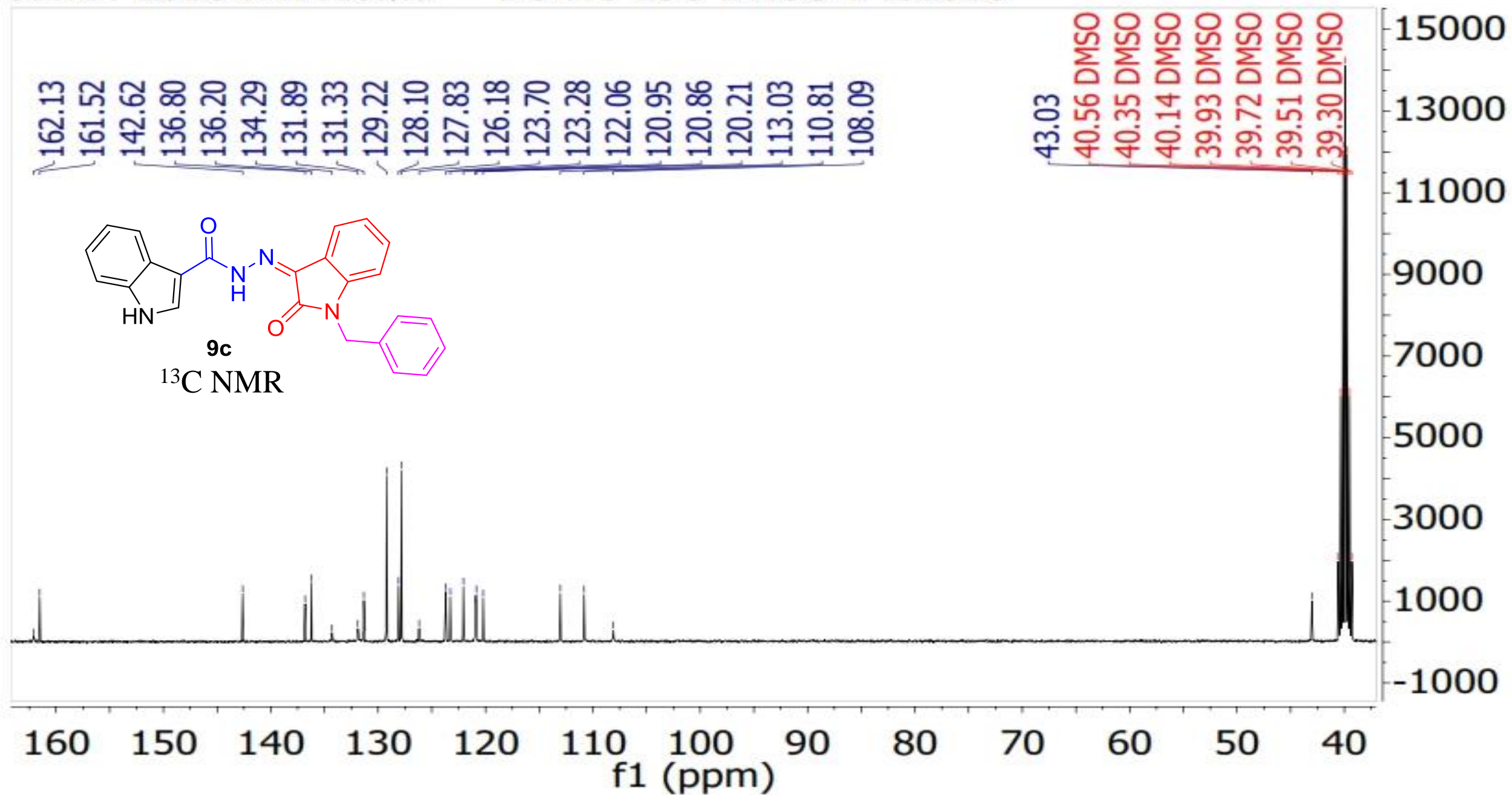

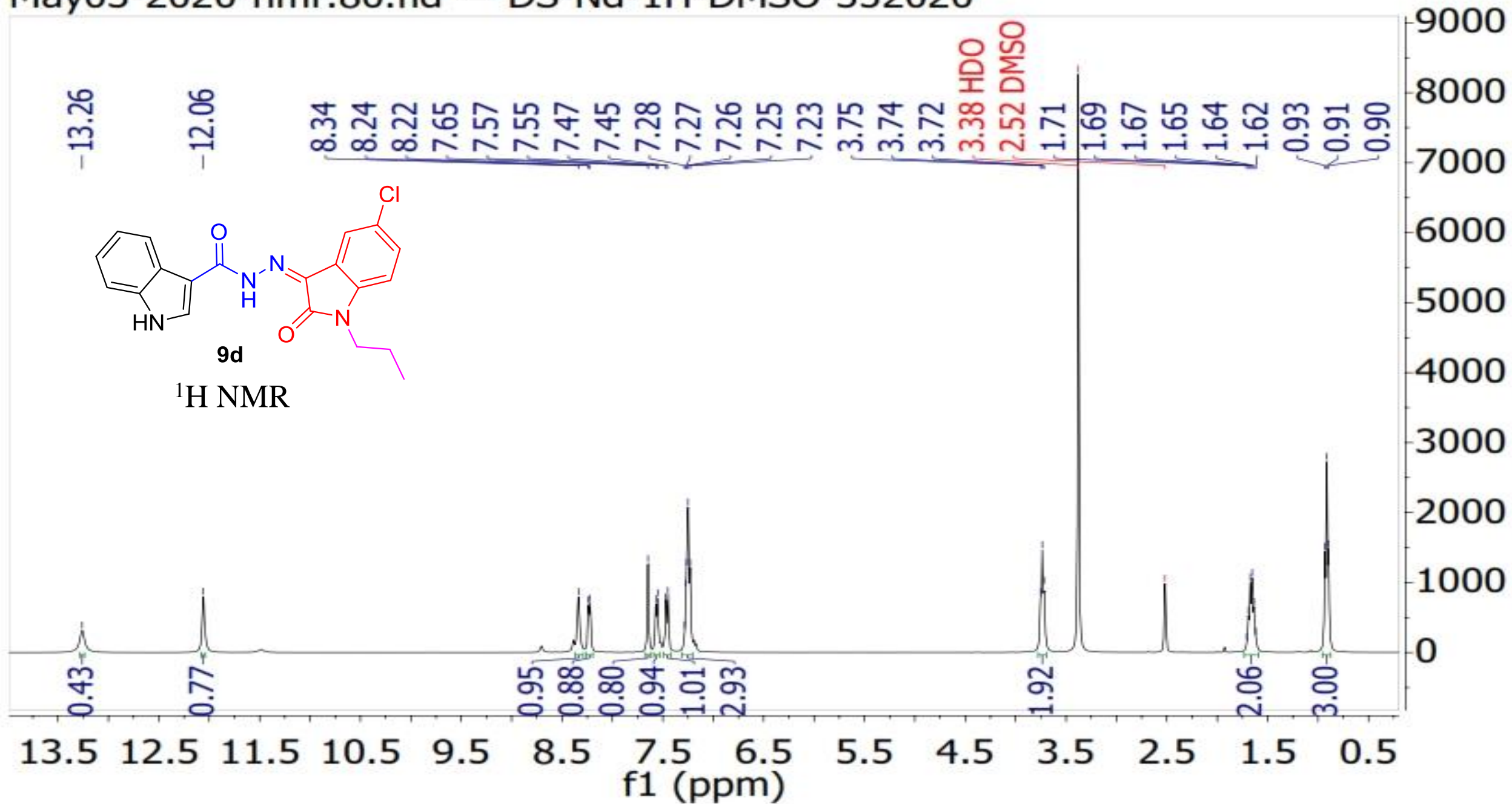

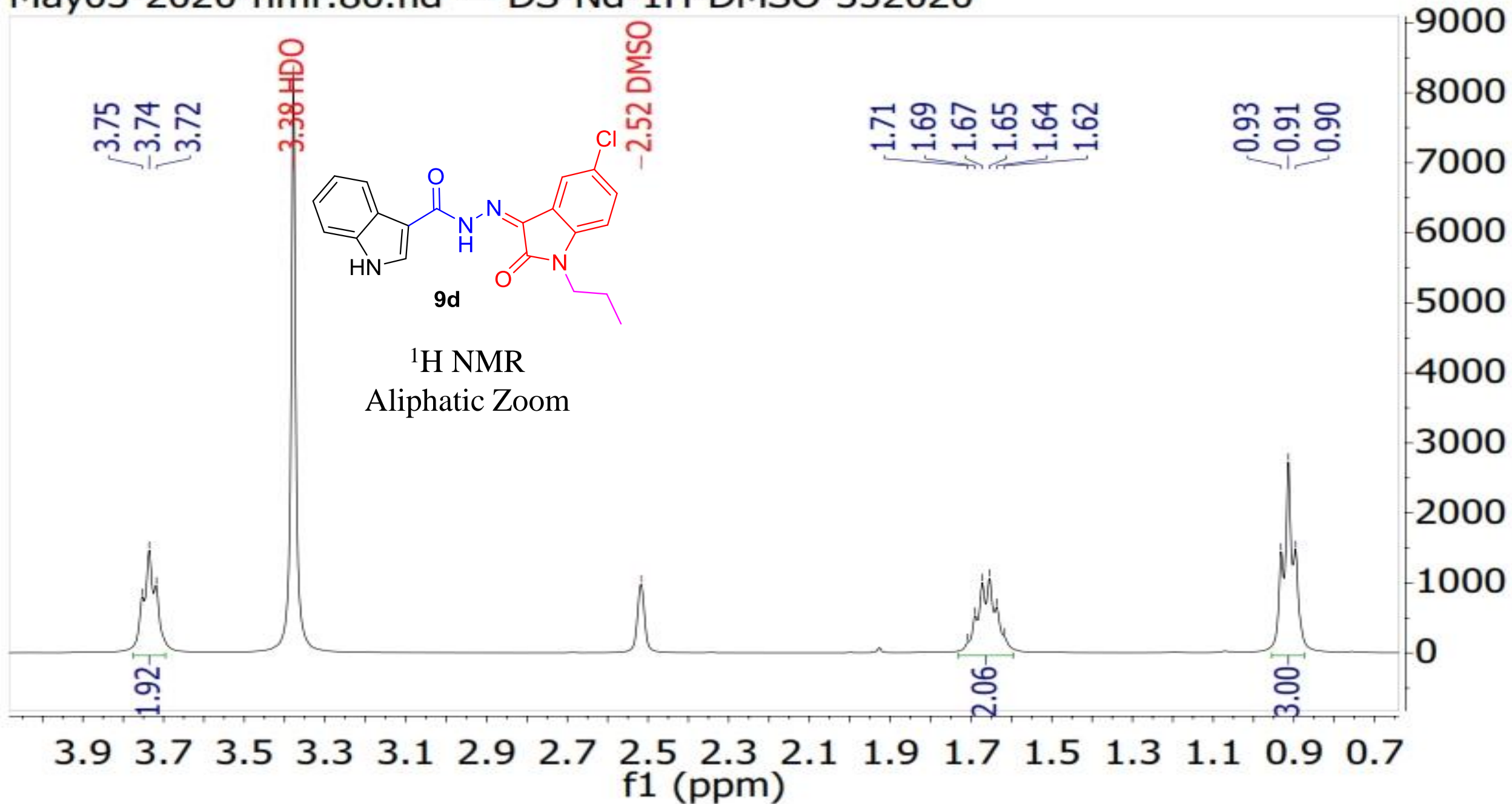

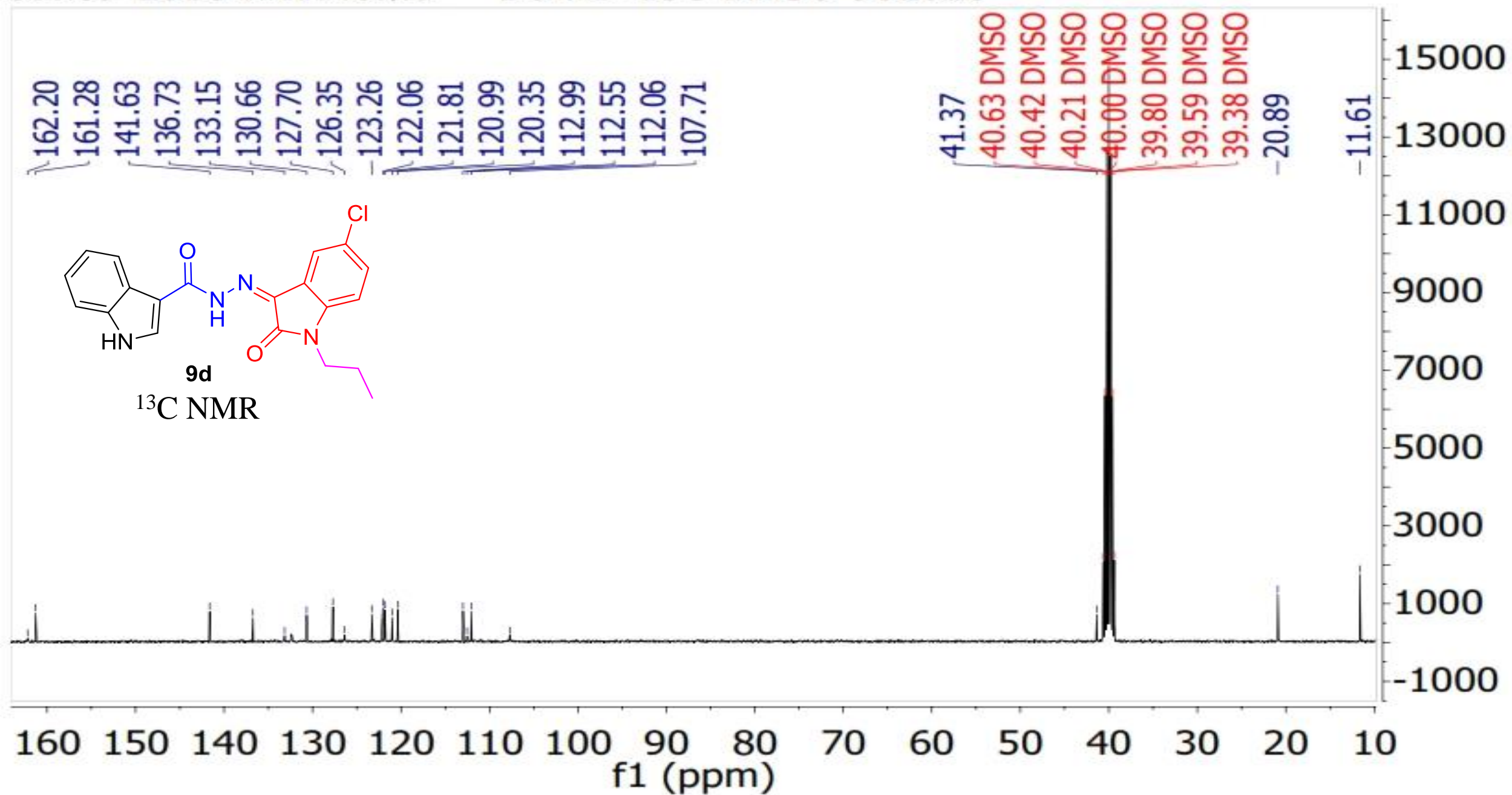

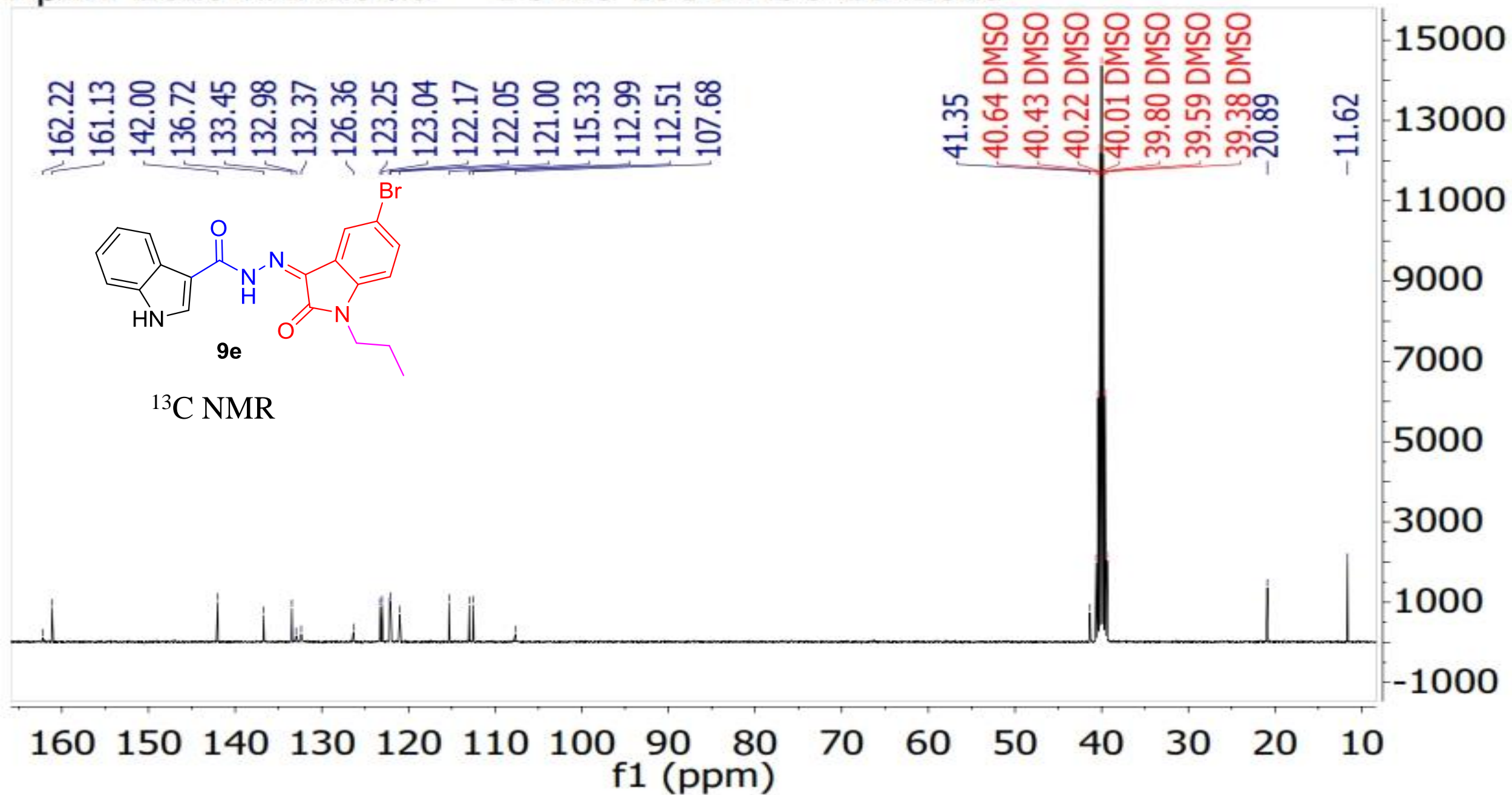

Supplement: Supplemental Material [file IENZ_A_1862100_SM0360.pdf]
